# Neophytadiene, a Plant Specialized Metabolite, Mediates the Virus‐Vector‐Plant Tripartite Interactions

**DOI:** 10.1002/advs.202416891

**Published:** 2025-04-03

**Authors:** Xiao‐bin Shi, Hao Yue, Yan Wei, Evan L. Preisser, Pei Wang, Jiao Du, Ji‐xing Xia, Kai‐long Li, Xin Yang, Jian‐bin Chen, Song‐bai Zhang, Zhan‐hong Zhang, Xu‐guo Zhou, De‐yong Zhang, Yong Liu

**Affiliations:** ^1^ Institute of Plant Protection Hunan Academy of Agricultural Sciences Yuelushan Laboratory Changsha 410125 China; ^2^ Laboratory of Agricultural and Forestry Biosecurity MOA Key Lab of Pest Monitoring and Green Management College of Plant Protection China Agricultural University Beijing 100193 China; ^3^ Department of Biological Sciences University of Rhode Island Kingston RI 02881 USA; ^4^ State Key Laboratory of Vegetable Biobreeding Department of Plant Protection Institute of Vegetables and Flowers Chinese Academy of Agricultural Sciences Beijing 100081 China; ^5^ Institute of Vegetable Hunan Academy of Agricultural Sciences Yuelushan Laboratory Changsha 410125 China; ^6^ Department of Entomology School of Integrative Biology College of Liberal Arts & Sciences University of Illinois Urbana‐Champaign Urbana IL 61801 USA

**Keywords:** *Bemisia tabaci*, chlorophyll degradation, neophytadiene, odorant‐binding protein, tomato chlorosis virus

## Abstract

While interactions between viruses and their vectors, as well as between viruses and host plants, have been extensively studied, the genetic mechanisms underlying tripartite interactions remain largely unknown. In this study, phenotypic assays are integrated with molecular biology and functional genomic approaches to elucidate the tripartite interactions involving tomato chlorosis virus (ToCV), a major threat to tomato production worldwide, the whitefly, *Bemisia tabaci*, an insect vector, and host plants. ToCV infection induces the production of a chlorophyll degradation product that acts as a volatile attractant for whiteflies. Furthermore, the suppression of *Lhca4*, a gene encoding subunit of light‐harvesting complex I in host plants, by the P9 protein of ToCV leads to chlorophyll degradation and neophytadiene biosynthesis. Overexpression of *Lhca4* reduced chlorophyll production and ToCV infection. Furthermore, OBP2, an odorant‐binding protein from *B. tabaci*, capable of binding to neophytadiene is identified. Suppression of *BtOBP2* impaired vector's subsequent preference for ToCV‐infected plants. The results not only reveal the genetic underpinnings, including ToCV P9, host plant Lhca4, and whitefly BtOBP2, governing the virus‐vector‐plant interactions, but also highlight neophytadiene, a specialized metabolite in host plants, as a mediator of intricate multitrophic interactions, suggesting new avenues for managing plant virus vectored by insects.

## Graphical abstract

Tomato chlorosis virus (ToCV) infection induces production of neophytadiene, a volatile resulting from chlorophyll degradation that is highly attractive to whiteflies. OBP2, an odorant‐binding protein from insect vector *B. tabaci*, exhibits a strong binding affinity for neophytadiene. The results identify key targets in virus‐vector‐plant interactions, and open new avenues for managing plant virus vectored by insects.

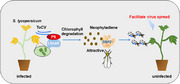

## 1 Introduction

Vector transmission is an essential step in the infection cycle of most plant viruses. Many vector‐borne plant viruses can manipulate both their vectors and host plants to facilitate virus transmission.^[^ ^1^, ^2^ ^]^ The interactions between viruses and their vectors, as well as between viruses and host plants, have been extensively studied.^[^ ^3^, ^4^ ^]^ Plant viruses manipulate host plants and insect vectors through diverse mechanisms, including altering plant volatile emissions, modulating vector feeding behavior, and suppressing host immune responses.^[^ ^5^, ^6^ ^]^ However, the genetic basis underlying these three‐way interactions remains largely unknown.

ToCV poses a significant threat to global tomato production,^[^ ^7^ ^]^ having spread to nearly 40 countries and territories, with the potential for total yield loss.^[^ ^8^ ^]^ It is transmitted in a semi‐persistent manner by *B. tabaci* and several other insects.^[^ ^9^ ^]^ *B. tab*aci is highly polyphagous and capable of transmitting a variety of plant viruses.^[^ ^10^ ^]^ While previous research has indicated that ToCV infection attracts whitefly,^[^ ^11^ ^]^ the molecular mechanisms underlying these changes remain largely unknown.

An early sign of ToCV infection is the yellowing of leaves, accompanied by irregular chlorosis.^[^ ^7^ ^]^ Several viruses have been shown to trigger changes in chloroplast physical structure and photoprotective processes.^[^ ^12^, ^13^, ^14^ ^]^ For instance, cabbage leaf curl virus upregulates the expression of genes involved in chlorophyll degradation.^[^ ^15^ ^]^ Lhca proteins are integral components of the photosynthetic apparatus in plants, playing a crucial role in the stability of chlorophyll and its degradation during leaf senescence and stress conditions.^[^ ^16^ ^]^ Neophytadiene, a volatile compound, is produced as a result of chlorophyll degradation in many plant species.^[^ ^17^ ^]^ This volatile compound is highly attractive to whiteflies; previous research has demonstrated that plants infected with TYLCV exhibit increased production of neophytadiene.^[^ ^18^ ^]^

While virus infection mediates plant volatile and plant defense, including jasmonic acid (JA) and salicylic acid (SA), along with the related genes such as proteinase inhibitor II (*PI II*), lipoxygenase (*LOX*), pathogenesis‐related 1 (*PRI*), and non‐expressor of PR genes 1 (*NPRI*) to attract insect vectors,^[^ ^19^, ^20^ ^]^ the identification of key protein that bridge the interactions among viruses, vectors, and plants is only beginning to be characterized. The successful application of structural cuticle proteins (CPs) might have dual roles in the virus–vector interactions and silencing CP genes provide hope for development of a new control strategies for viruses and their vectors.^[^ ^21^ ^]^ The 2b protein of cucumber mosaic virus (CMV) inhibits JA signaling, thereby enhancing the fecundity of its aphid vector *Myzus persicae*,^[^ ^22^ ^]^ while the NIa‐Pro protein of turnip mosaic virus (TuMV) promotes *M. persicae* reproduction by inhibiting callose formation.^[^ ^23^ ^]^ Similarly, the C2 protein of tomato yellow leaf curl virus (TYLCV) improves survival of its vector, *B. tabaci*, by inhibiting JA‐mediated plant defenses,^[^ ^24^ ^]^ and the βC1 protein of tomato yellow leaf curl China virus (TYLCCNV) suppresses the synthesis of terpenes that repel *B. tabaci*.^[^ ^17^ ^]^ The NS protein of tomato spotted wilt orthotospovirus (TSWV) reduces monoterpene biosynthesis, thereby enhancing the survival and transmission efficiency of its thrips vector (*Frankliniella occidentalis*).^[^ ^25^ ^]^

Odorant‐binding proteins (OBPs), small and soluble proteins located in the insect antennae, play a pivotal role in the detection and processing of volatile compounds. These proteins can modulate plant responses through the selective binding of volatiles released during virus infections.^[^ ^26^ ^]^ OBP‐mediated olfactory mechanisms have driven research into how OBPs recognize and respond to volatile cues. OBPs influence the attraction of insect vectors to infected hosts, which in turn facilitates the spread of viruses.^[^ ^27^ ^]^ Understanding the underlying mechanisms of OBP function offers novel insights into vector control strategies and virus management. By targeting OBP pathways, we could potentially disrupt the ability of insects to locate infected hosts, thereby mitigating virus transmission.

Here, we integrated techniques from ecology and molecular biology to comprehensively elucidate the tripartite interactions among ToCV, *B. tabaci*, and host plants. Specifically, we conducted whitefly preference tests and protein interaction test to determine mechanism of neophytadiene release, which is mediated by chlorophyll degradation following ToCV infection, thereby attracting whiteflies. Neophytadiene, a specialized metabolite in host plants, mediates these intricate multitrophic interactions, suggesting new avenues for managing this devastating virus.

## 2 Results

### 2.1 ToCV Infection Induced Neophytadiene Production and Attracted Whiteflies

**Figure 1:**
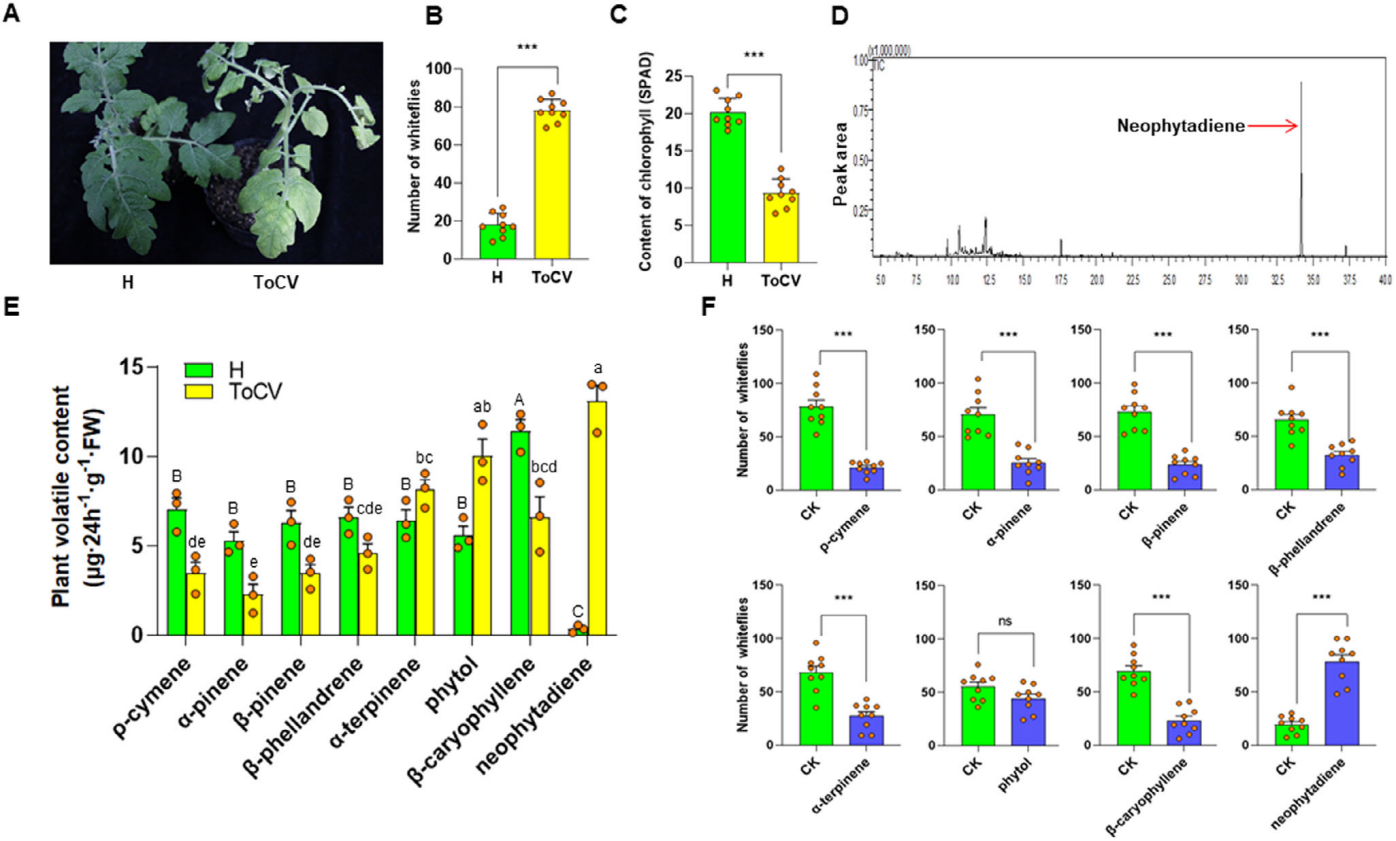
Neophytadiene plays an important role in attracting *Bemisia tabaci* whiteflies. A) Leaves of control (H) and ToCV‐infected (ToCV) tomato plants. B) Whitefly preference for control (green bars) versus ToCV‐infected (yellow bars) tomato plants (*n* = 9, t‐test). C) Chlorophyll content in control and ToCV‐infected tomato plants (*n* = 9, t‐test). D) Chromatographic peak of neophytadiene. E) Volatiles generated from control and ToCV‐infected tomato plants (*n* = 3, GLM). F) Whitefly preference for plant volatiles versus control (solvent only) (*n* = 9, GLM). Bars in each graph depict mean ± SE; orange dots are individual replicates. ***P < 0.001 (t‐test). Different uppercase letters indicate significant differences on volatile content with control plants (*P* < 0.05); Different lowercase letters indicate significant differences on volatile content with ToCV‐infected plants (*P* < 0.05).

ToCV infection resulted in leaf chlorosis (**Figure** **1**A), and infected plants attracted 4.3 times more whiteflies than control plants (*p* < 0.001; Figure 1B). The chlorophyll content of infected leaves was 54% lower than that in control leaves (*p* < 0.001; Figure 1C). Plant volatile content showed significant differences among different volatile types (*p* < 0.001), and the interaction between ToCV infection and volatile type (*p* < 0.001), but showed no difference between H and ToCV infection (*p* = 0.322) (Figure 1E). Compared to healthy plants, ToCV‐infected plants exhibited a reduction in the production of the volatile compounds ρ‐cymene by 51%, α‐pinene (57%), β‐pinene (45%), and β‐caryophyllene (42%). In contrast, phytol production increased by 80%, and neophytadiene levels were 37 times higher (Figure 1D,E; Figure S1, Supporting Information). A Y‐tube olfactory choice assay was conducted to evaluate the impact of each volatile on whitefly preference. Whitefly preference showed a significant difference between CK and different volatile treatment (*p* < 0.001), and the interaction between volatile treatment and volatile type (*p* < 0.001), but showed no difference among different volatile types (*p* = 0.997) (Figure 1F). The compounds ρ‐cymene, α‐pinene, β‐pinene, β‐phellandrene, α‐terpinene, and β‐caryophyllene significantly repelled whiteflies, while phytol did not influence whitefly preference. Neophytadiene was the only compound that attracted *B. tabaci*, with the significant change of quantity.

### 2.2 ToCV Infection Modified Whitefly Feeding Behavior by Manipulating Plant Defense

**Figure 2:**
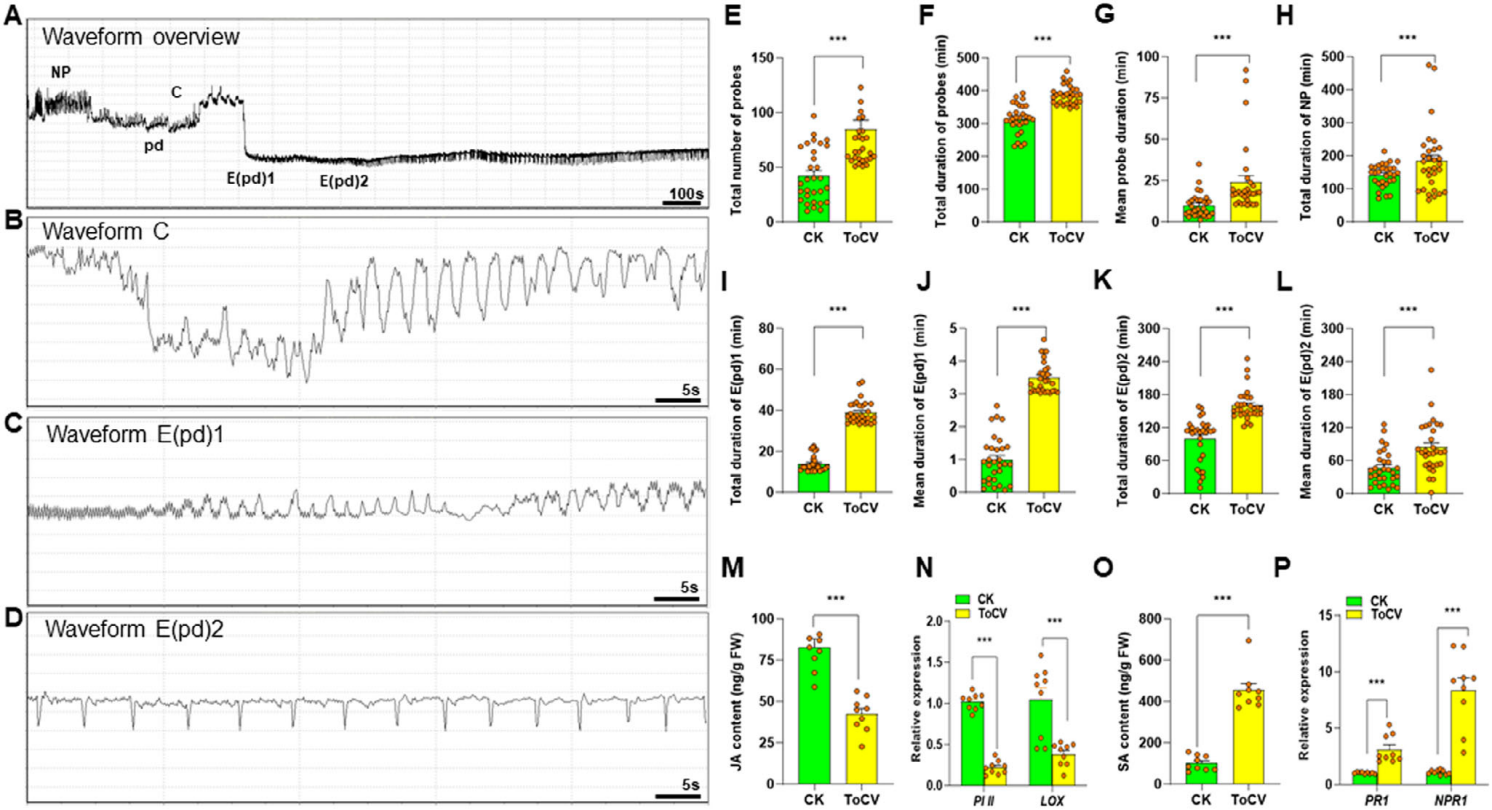
Impact of ToCV infection on whitefly feeding behavior. A‐D) Representative EPG waveform patterns generated by *B. tabaci* feeding on host plants. E) Total number of probes of control (H; green bar) and ToCV‐infected (ToCV; yellow bar) tomato plants (*n* = 9, t‐test). F) Total duration of probes (*n* = 9, t‐test). G) Mean probe duration (*n* = 9, t‐test). H) Total duration of non‐probing behavior (NP) (*n* = 9, t‐test). I) Total duration of E(pd)1, salivation (viral transmission phase) (*n* = 9, t‐test). J) Mean duration of E(pd)1 (*n* = 9, t‐test). K) Total duration of E(pd)2, feeding (viral acquisition phase) (*n* = 9, t‐test). L) Mean duration of E(pd)2 (*n* = 9, t‐test). M) Jasmonic acid content of control and ToCV‐infected tomato plants (*n* = 9, t‐test). N) Relative expression of *PI II* and *LOX* in control and ToCV‐infected tomato plants (*n* = 9, t‐test). O) Salicylic acid content of control and ToCV‐infected tomato plants (*n* = 9, t‐test). P) Relative expression of *PR1* and *NPR1* in control and ToCV‐infected tomato plants (*n* = 9, t‐test). In Figure 2e–m, and o, ***P < 0.001 (Tukey's HSD). In Figure 2n,p, lower‐case letters indicate significant differences at *P* < 0.05 (Tukey's HSD). Bars in each graph depict mean ± SE; orange dots are individual replicates.

We evaluated the feeding behavior of *B. tabaci* on both virus‐free and ToCV‐infected plants using an electrical penetration graph (EPG; **Figure** **2**A–D). Over an 8 *h* recording period, whiteflies feeding on ToCV‐infected plants exhibited nearly double the number of probes (Figure 2E) and spent 23% more total time probing (Figure 2F) compared to those on virus‐free plants. Furthermore, they engaged in more phloem feeding on infected plants, spending nearly three times as much time salivating (E(pd)1; Figure 2I,J) and almost twice as long ingesting phloem (E(pd)2; Figure 2K,L). These findings indicate that *B. tabaci* fed more avidly on ToCV‐infected plants than virus‐free controls.

Due to the influence of plant defense mechanisms on whitefly feeding behavior, we evaluated the JA and SA signaling pathways in both virus‐free and ToCV‐infected plants. The JA levels in the infected plants were found to be 49% lower than those in the control group, and the expression of the associated *PI II* and *LOX* genes was significantly suppressed (all *p* < 0.001; Figure 2M,N). In contrast, the SA levels in the infected plants were 4.5‐fold higher, and the expression of the associated *PR1* and *NPR1* genes were significantly induced (all *p* < 0.001; Figure 2O,P). These findings indicate that ToCV‐induced alterations in plant defense responses may have modified whitefly feeding behavior.

### 2.3 ToCV P9 Induced Neophytadiene Release

**Figure 3:**
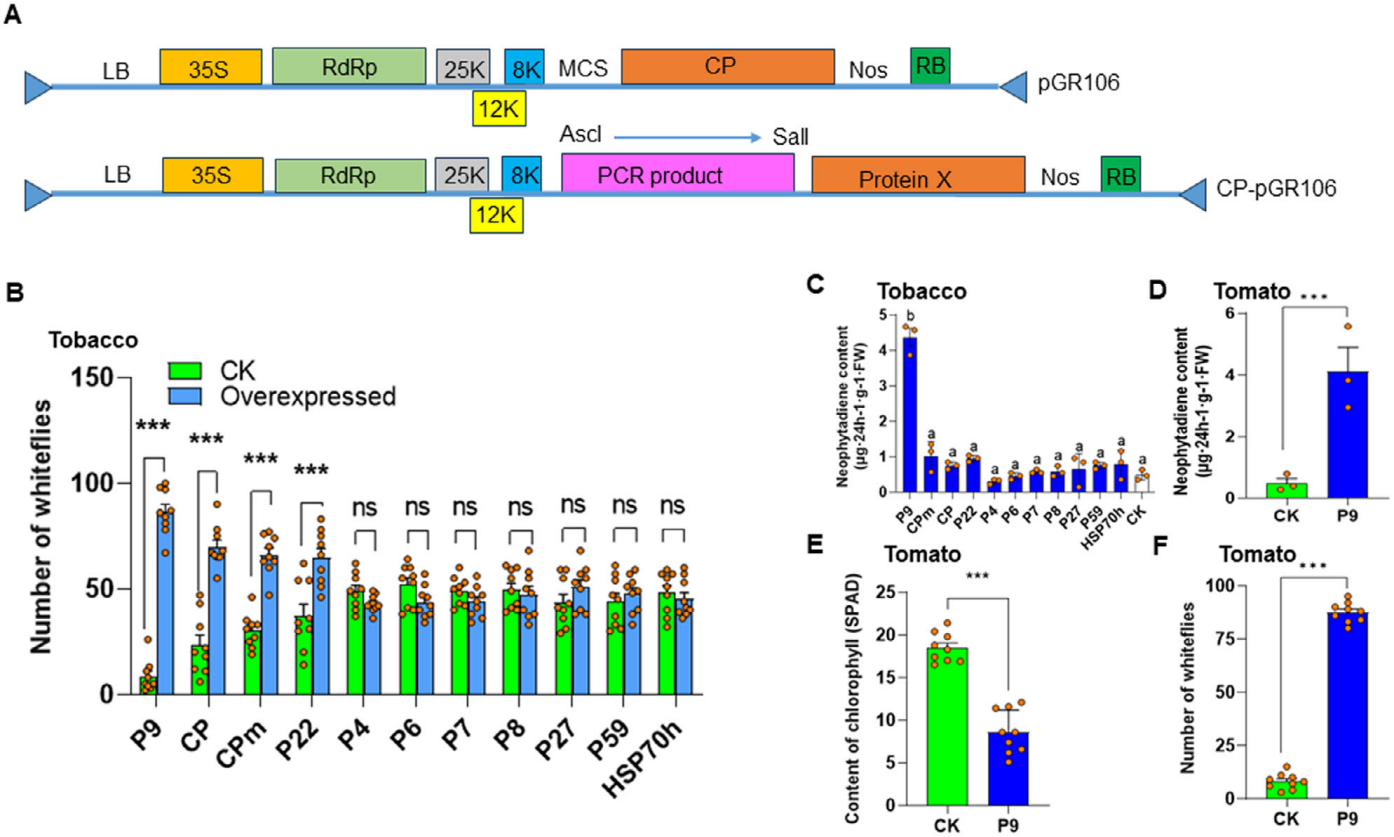
The ToCV P9 protein induces neophytadiene production. A) Process by which the 11 ToCV proteins were transiently overexpressed in tobacco or tomato plants. B) Number of whiteflies on control (CK; green bars) or ToCV‐protein‐overexpressing (overexpressed; blue bars) tobacco plants (*n* = 9, GLM). C) Neophytadiene content in ToCV‐protein‐overexpressing tobacco plants (*n* = 3, Tukey's HSD post‐hoc test). D) Neophytadiene content in control or P9‐overexpressing tomato plants (*n* = 3, t‐test). E) Chlorophyll content in control or P9‐overexpressing tomato plants (*n* = 9, t‐test). F) Number of whiteflies on control or overexpressing tomato plants (*n* = 9, t‐test). ***P < 0.001. Lower‐case letters indicate significant differences at *P* < 0.05. Bars in each graph depict mean ± SE; orange dots are individual replicates.

To identify the protein responsible for mediating neophytadiene release, all 11 ToCV proteins were transiently overexpressed in tobacco plants (**Figure** **3**A). Whitefly preference for control versus overexpressed tobacco plants was then compared. Whitefly preference showed a significant difference between CK and overexpressed plants (*p* < 0.001), and the interaction between overexpressing treatment and different viral protein (*p* < 0.001), but showed no difference among different viral protein‐overexpressing plants (*p* = 0.954) (Figure 3B). Whiteflies preferred plants overexpressing the proteins P9, CPm, CP, and P22 (Figure 3B), and neophytadiene levels were higher only in plants overexpressing the P9 protein (*p* < 0.001; Figure 3C).

To confirm the relationship between the P9 protein and the release of neophytadiene, we overexpressed the P9 protein in tomato plants. The neophytadiene content was found to be 8.4‐fold higher in P9‐overexpressing tomato plants compared to control plants (*p* < 0.001; Figure 3D). Neophytadiene is a product of chlorophyll degradation (Figure S2, Supporting Information), and the chlorophyll content in the overexpressing plants was 54% lower (*p* < 0.001; Figure 3E). Additionally, whiteflies demonstrated a tenfold preference for P9‐overexpressing plant over control plants (*p* < 0.001; Figure 3F). The consistent results observed in both tobacco and tomato plants suggest that the P9 protein enhances neophytadiene production through chlorophyll degradation.

### 2.4 P9 Protein Interacts with Light‐Harvesting Complex (LHC) I Subunit Lhca4

**Figure 4:**
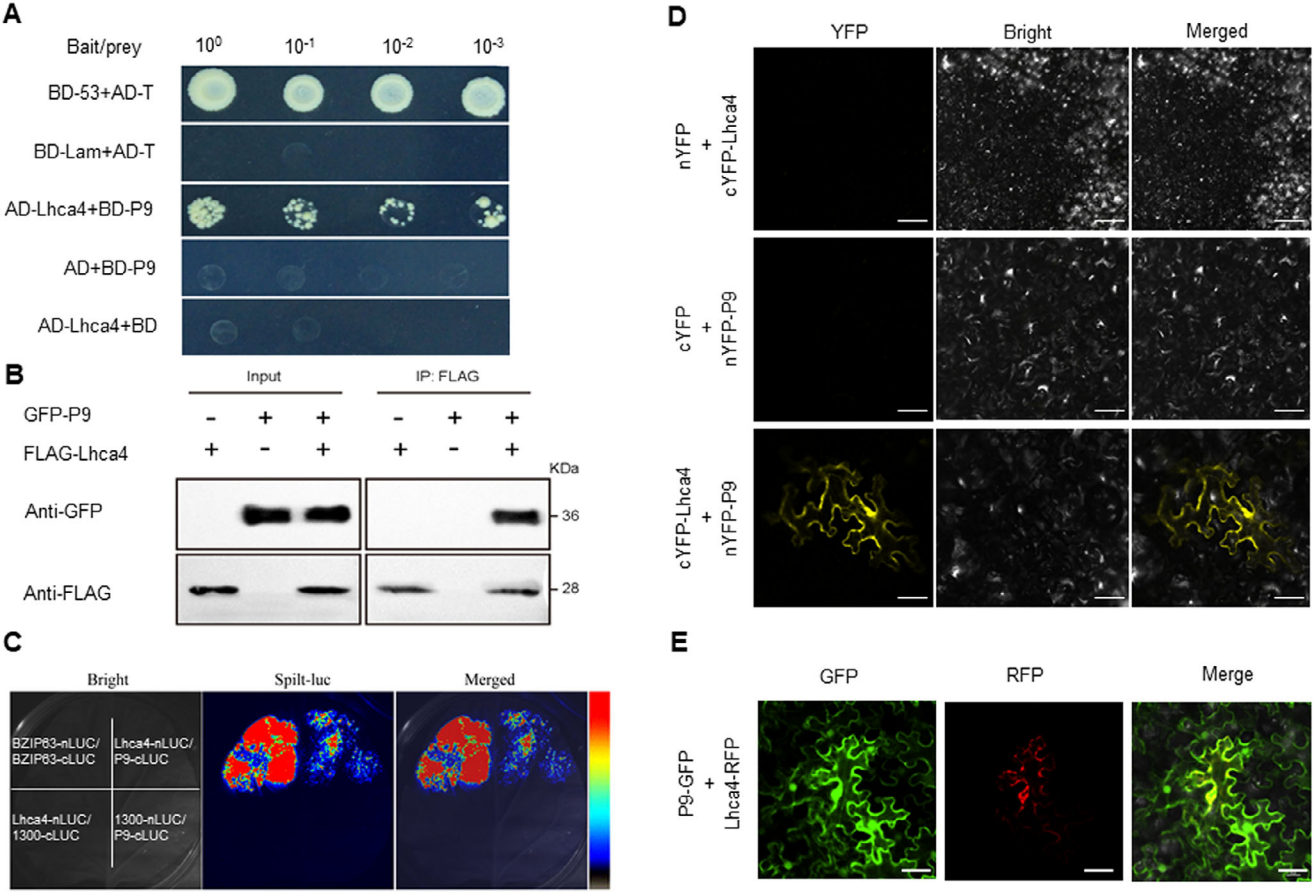
The ToCV P9 protein interacts with the host plant Lhca4 protein. A) Interactions between ToCV P9 and Lhca4 identified by yeast two‐hybrid assays. pGBKT7‐53 (BD‐53) and pGADT7‐T (AD‐T) vectors were used as a positive control, while pGBKT7‐Lam (BD‐Lam) and pGADT7‐T vectors were used as a negative control. B) Lhca4‐FLAG was co‐expressed with ToCV P9‐GFP in tobacco. Total protein was extracted and immunoprecipitated with anti‐FLAG magnetic beads. Western blots were carried out using monoclonal anti‐FLAG or anti‐GFP antibodies to explore FLAG‐tagged proteins or co‐immunoprecipitated GFP‐tagged protein, respectively. C) Dual luciferase assay of P9 and Lhca4. P9 fused to the LUC C‐terminal region and Lhca4 fused to the LUC N‐terminal region were co‐transformed into tobacco leaves. D) BiFC assay of the P9 and Lhca4 interaction. P9 fused to the YFP N‐terminal region and Lhca4 fused to the YFP C‐terminal region were co‐transformed into tobacco leaves. E) Co‐localization of Lhca4‐mCherry with P9‐GFP in tobacco leaf cells.

To investigate the effects of the P9 protein on tomato plants, yeast two‐hybrid assays were conducted (Figure S3, Supporting Information). These assays demonstrated that P9 directly interacts with Lhca4 (**Figure** **4**A), a subunit of the light‐harvesting complex I (LHC I). Coimmunoprecipitation (*C* *o*‐*I* *P*) assays indicated that Lhca4 was strongly pulled down by P9 in vitro (Figure 4B). Subsequently, firefly luciferase complementation imaging (LCI) was performed in tobacco leaves to access the P9‐Lhca4 interaction in planta. Luciferase activity was reconstituted when P9‐nLUC and cLUC‐Lhca4 were co‐expressed in tobacco leaves (Figure 4C). An *Agrobacterium tumefaciens* solution containing various fluorescent protein constructs was then infiltrated into pairs of tobacco leaves. After 48 *h* of inoculation, tobacco leaf cells were examined using laser confocal microscopy, which provided further confirmation of the interactions between P9 and Lhca4 (Figure 4D). Finally, transiently overexpressed P9 revealed P9 and Lhca4 colocalize in both the cytoplasm and nuclei of the cells. (Figure 4E; Figure S4, Supporting Information).

### 2.5 Overexpression of *Lhca4* Increased Chlorophyll Accumulation and Reduced ToCV Infection

**Figure 5:**
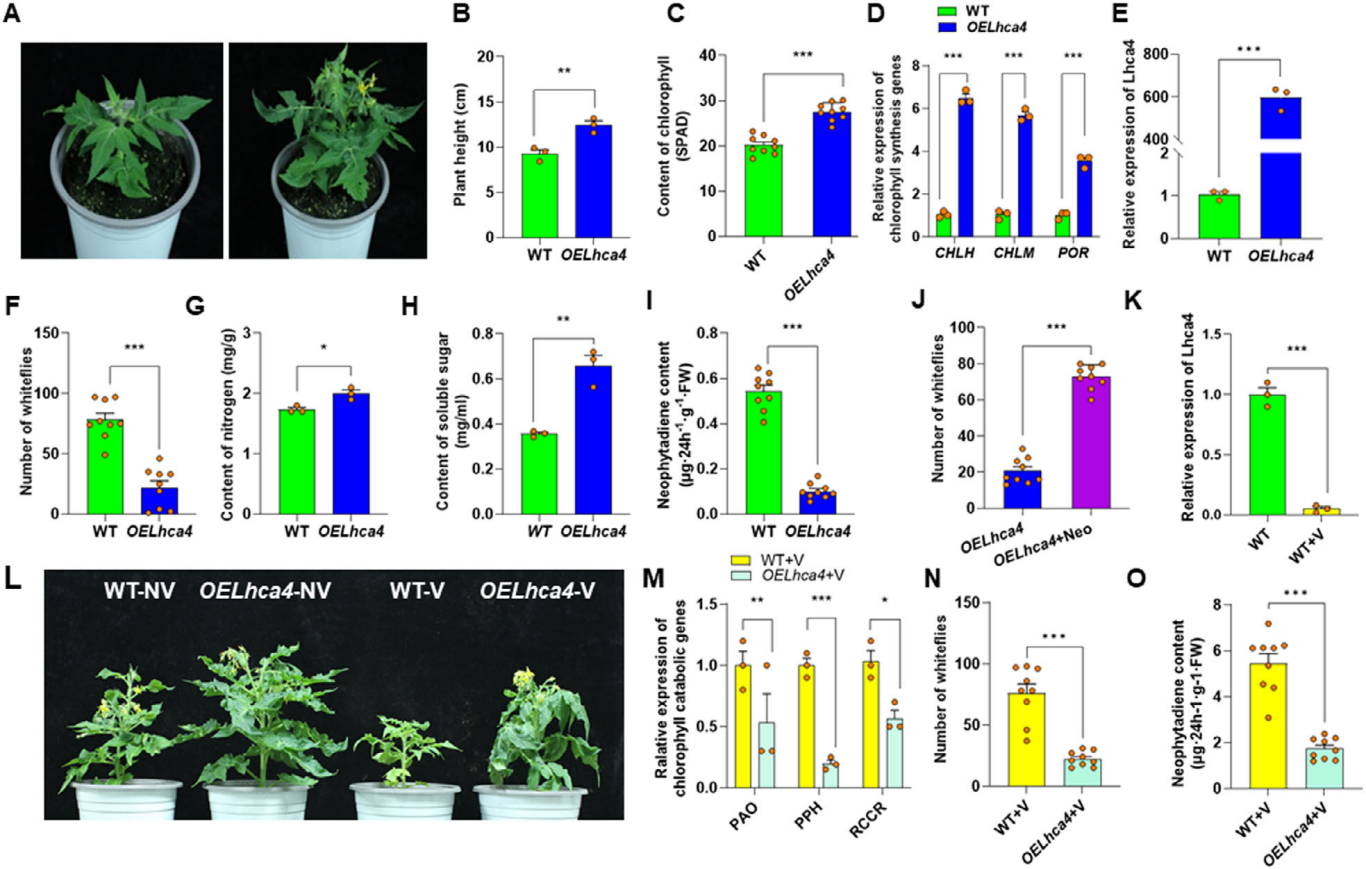
Lhca4‐overexpressed tomato plants had more chlorophyll, attracted fewer whiteflies, and were less affected by ToCV. A) Control (left) and Lhca4‐overexpressed (right) tomato plants. B) Height of control (WT; green bar) and Lhca4‐overexpressed (*OELhca4*; blue bar) plants (*n* = 3, t‐test). C) Chlorophyll content in control and Lhca4‐overexpressed plants (*n* = 9, t‐test). D) Relative expression of CHLH, CHLM, and POR in control and Lhca4‐overexpressed plants (*n* = 3, t‐test). E) Relative expression of Lhca4 in control and Lhca4‐overexpressed plants (*n* = 3, t‐test). F) Whitefly preference for control versus Lhca4‐overexpressed plants (*n* = 9, t‐test). G) Nitrogen content in control and Lhca4‐overexpressed plants (*n* = 3, t‐test). H) Soluble sugar content in control and Lhca4‐overexpressed plants (*n* = 3, t‐test). I) Neophytadiene content in control and Lhca4‐overexpressed plants (*n* = 9, t‐test). J) Whitefly preference for Lhca4‐overexpressed tomato plants with (*OELhca4*; blue bar) or without (*OELhca4*+Neo; purple bar) neophytadiene (*n* = 9, t‐test). K) Relative expression of Lhca4 in control and ToCV‐infected plants (*n* = 3, t‐test). L) Effect of ToCV infection on control or *OELhca4* plants. Left pair: healthy control and *OELhca4* plants; right pair: ToCV‐infected control and *OELhca4* plants. M) Relative expression of the chlorophyll catabolic genes PAO, PPH, and RCCR in control (yellow bars) or *OELhca4* (light green bars) plants after ToCV infection (*n* = 3, t‐test). N) Whitefly preference for control versus Lhca4‐overexpressed plants after ToCV infection (*n* = 9, t‐test). O) Neophytadiene content in control and Lhca4‐overexpressed plants after ToCV infection (*n* = 9, t‐test). *P < 0.05, **P < 0.01, ***P < 0.001. Bars in each graph depict mean ± SE; orange dots are individual replicates.

Lhca4 is a photosystem I antenna protein that has been reported to red‐shift chlorophyll d absorption.^[^ ^28^ ^]^ We explored its function by generating overexpressed *Lhca4* (*OELhca4*) tomato plants. The *OELhca4* plants exhibited a greener appearance compared to wild‐type plants (**Figure** **5**A), and were 34% taller (*p* = 0.008; Figure 5B), with a 37% increase in chlorophyll content (*p* < 0.001; Figure 5C). Assays revealed an induction of the chlorophyll synthesis genes, including *CHLH*, *CHLM*, and *POR*, in *OELhca4* plants (*p* < 0.001; Figure 5D), which demonstrated a 5.8‐fold increase in overall gene expression compared to wild‐type tomato plants (*p* < 0.001; Figure 5E). Furthermore, whiteflies strongly preferred wild‐type plants over *OELhca4* plants (*p* < 0.001; Figure 5F), even though *OELhca4* plants contained higher levels of nitrogen and soluble sugar (*p* = 0.016 and *p* = 0.003, respectively; Figure 5G,H). Neophytadiene content of *OELhca4* plants was reduced by 80% compared to wild‐type plants (*p* < 0.001; Figure 5I), and neophytadiene‐sprayed *OELhca4* plants attracted more whiteflies than water‐sprayed *OELhca4* plants (Figure 5J).

*Lhca4* gene expression is closely linked to chlorophyll content.^[^ ^29^ ^]^ As anticipated, ToCV infection suppressed *Lhca4* gene expression (Figure 5K). Inoculating *OELhca4* plants with ToCV resulted in mild chlorosis and a reduction in plant height; however, both virus‐free and ToCV‐infected *OELhca4* plants remained taller than the wild‐type counterparts (Figure 5L; Figure S5, Supporting Information). Compared to ToCV‐infected wild‐type plants, ToCV‐infected *OELhca4* plants exhibited a greater decrease in the expression of genes related to chlorophyll catabolic enzymes, such as *PAO*, *PPH*, and *RCCR* (Figure 5M). Additionally, whiteflies preferred ToCV‐infected wild‐type plants over ToCV‐infected *OELhca4* plants (Figure 5N), which had a lower neophytadiene content (Figure 5O). These findings underscore the role of Lhca4 in chlorophyll accumulation and demonstrate that ToCV infection reduces chlorophyll content in tomatoes through its interaction with Lhca4.

### 2.6 Overexpression of P9 Protein Increased Chlorophyll Degradation and Neophytadiene Release

**Figure 6:**
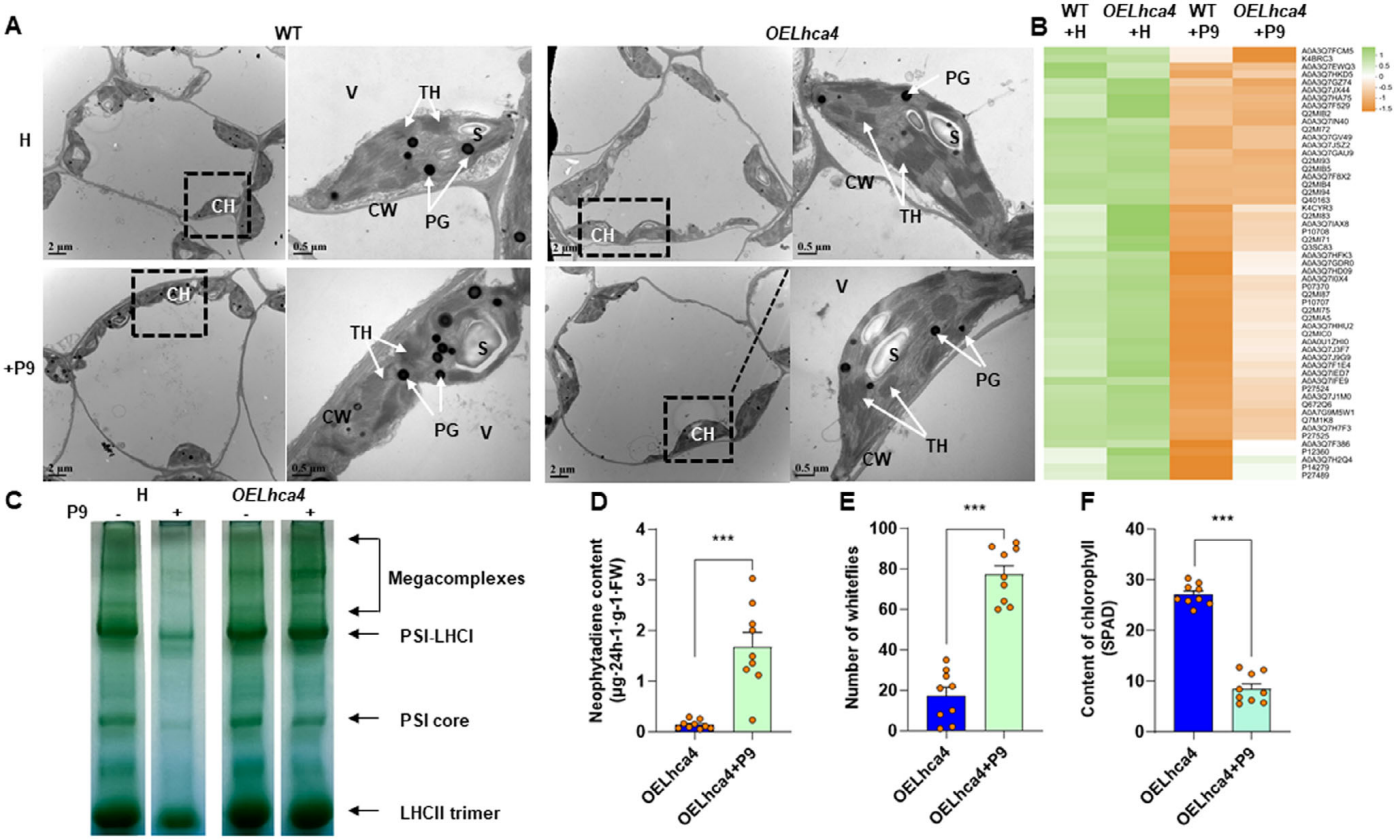
The ToCV P9 protein reduces the expression of the host plant Lhca4 protein. A) Electron microscopic observation of control (WT; left two columns) and overexpressed Lhca4 (*OELhca4*; right two columns) tomato leaves without (top row) or with (bottom row) P9 expression. Abbreviations:CW: Cell wall; CH: Chloroplast; PG: Plastoglobulus; TH: Thylakoid; S: Starch granules. B) Expression of photosystem‐related proteins in control (WT) and *OELhca4* tomato plants in the absence (left two green columns) or presence (right two orange columns) of P9 expression. C) Blue native PAGE analysis of thylakoid proteins from control and *OELhca4* plants with or without P9 expression. D) Neophytadiene content of *OELhca4* plants with or without P9 expression (*n* = 9, t‐test). E) Whitefly preference for *OELhca4* plants with or without P9 expression (*n* = 9, t‐test). F) Chlorophyll content of chlorophyll in *OELhca4* plants with or without P9 expression (*n* = 9, t‐test). ***P < 0.001. Bars in each graph depict mean ± SE; orange dots are individual replicates. The scale bar was 2 and 0.5 µm, respectively.

The P9 protein is unique to viruses in the genus *Crinivirus*, and no similar protein has been detected in other genera in the Closteroviridae.^[^ ^30^ ^]^ Up to now, however, the role of P9 in ToCV infection remains unexplored. Compared to wild‐type tomato plants, the chloroplasts of Lhca4‐overexpressing (“*OELhca4*”) tomato plants were oval in shape, contained more thylakoid with a clear and orderly structure, and had an increased number of starch granules (**Figure** **6**A). The *Lhca4* knockout plants (*KOLhca4*) were constructed as a control (Figure S6A, Supporting Information). Compared with the wild type plants, *KOLhca4* plants had the reduced chlorophyll content (Figure S6B,C, Supporting Information), increased neophytadiene content (Figure S6D, Supporting Information), and increased whitefly number (Figure S6E, Supporting Information). The knockout of *Lhca4* is detrimental to the growth of tomato plant, as the growth period is shortened, and all the seeds are stunted.

We next investigated the regulatory relationship between P9 and Lhca4 by transiently overexpressing the P9 protein in both wild‐type and *OELhca4* plants. P9‐inoculated plants exhibited enlarged chloroplasts, malformed starch granules, loose and disorganized inner capsules, as well as increases in both the volume and number of plastid globule; these symptoms were less apparent in *OELhca4* plants than wild‐type ones (Figure 6A). Notably, in P9‐inoculated tomato plants, the core subunits like PsbA (Q2MIC0), PsbC (A0A7G9M5W1), and PsbD (Q2MIA5) etc., and LHC proteins like Lhca4 (Q7M1K8), Lhcb3 (A0A3Q7HHU2) and Lhcb6 (P27524) etc. of PSI and PSII were down‐regulated relative to virus‐free plants (Figure 6B; Table S2, Supporting Information). Comparative proteomics identified a total of 5534 proteins, including 2944 differentially abundant proteins (DAPs), of which 1708 were up‐regulated and 1236 down‐regulated (Figure S7A and Table S3, Supporting Information). Function enrichment analysis revealed that DAPs induced by P9 overexpression were mainly involved in photosynthetic pathways (Figure S7B, Supporting Information).

We further explored the mechanism by which P9 regulates chlorophyll content through the inhibition of Lhca4 expression. Compared with the control, P9 expression caused a loose arrangement of stroma lamellae, irregular arrangement of grana lamellae, and malformed chloroplasts (Figure 6A). Lhca4 overexpression mitigated the P9‐induced damage to chloroplast ultrastructure in tomato (Figure 6A). Because the LHC binds the chlorophyll and anchors it to the thylakoid membrane,^[^ ^17^ ^]^ changes in PSI components that contain all Lhca subunits are critical determinants of chlorophyll content. Blue‐native polyacrylamide gel electrophoresis (BN–PAGE) was used to compare conditions before and after transient P9 overexpression. The PSI‐LHCI and PSI core bands were significantly reduced only in WT plants expressing P9, whereas this damage was significantly alleviated in *OELhca4* plants (Figure 6C). Furthermore, P9 overexpression increased neophytadiene content (*p* < 0.001; Figure 6D) and whitefly preference (*p* < 0.001; Figure 6E), while concurrently reducing chlorophyll content (*p* < 0.001; Figure 6F). These results indicate that P9 inhibition of Lhca4 affects PSI components and chloroplast ultrastructure, leading to chlorophyll degradation and increased neophytadiene production.

### 2.7 Neophytadiene Targets *B. Tabaci* BtOBP2 to Increase Vector Preference

**Figure 7:**
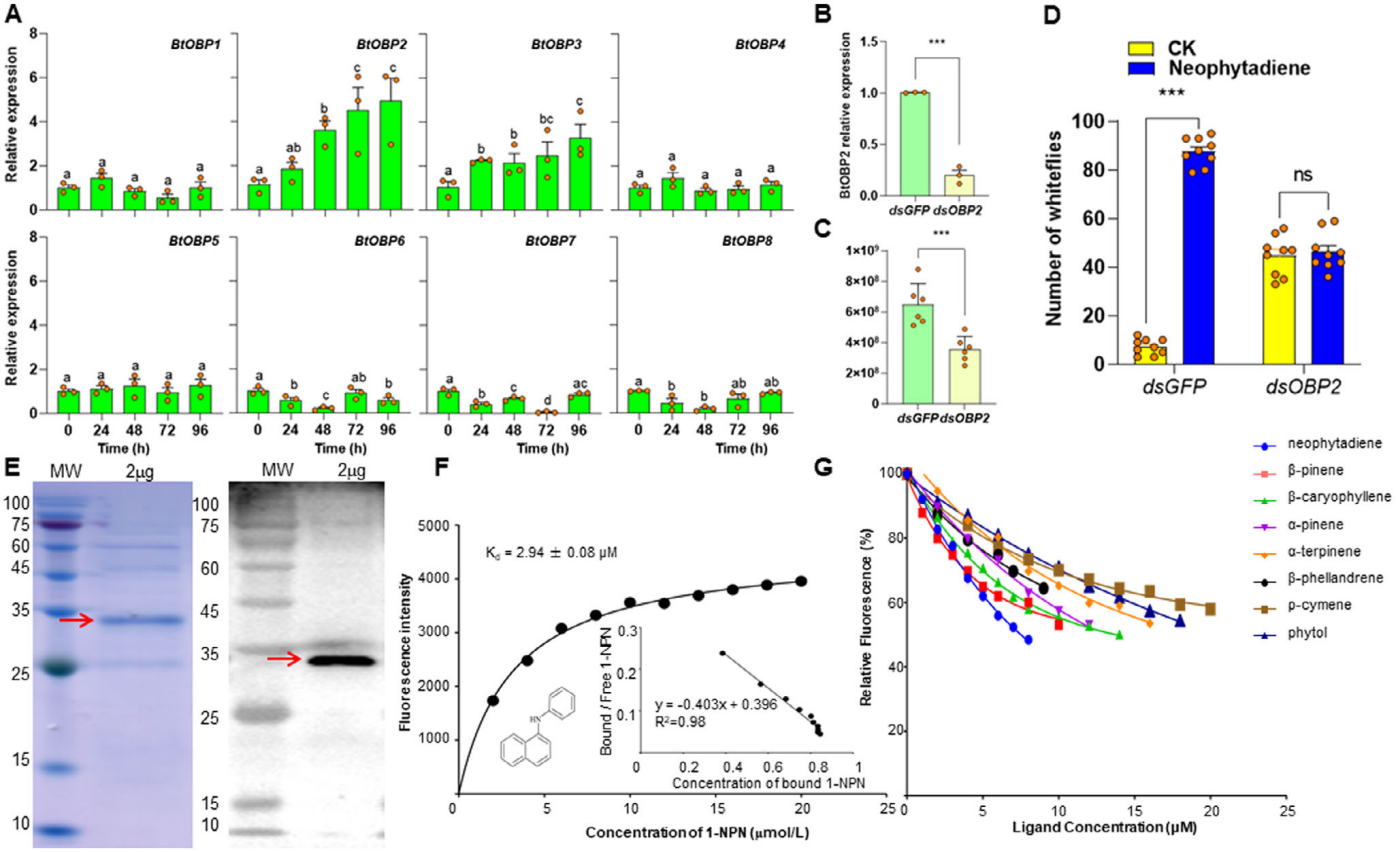
The whitefly BtOBP2 protein binds to neophytadiene and drives whitefly preference. A) Relative gene expression over time in BtOBP1‐8 following neophytadiene exposure (*n* = 3, Tukey's HSD post‐hoc test). B) Relative expression of *BtOBP2* in control (*dsGFP*) and *BtOBP2*‐silenced (*dsOBP2*) whiteflies (*n* = 3, t‐test). C) ToCV accumulation in control (*dsGFP*) and *BtOBP2*‐silenced (*dsOBP2*) whiteflies (*n* = 6, t‐test). D) Whitefly preference for control versus neophytadiene‐treated plants; left pair of bars are control whiteflies, right pair of bars are *BtOBP2*‐silenced whiteflies (*n* = 9, Two‐way ANOVAs). E) BtOBP2 expression by SDS‐PAGE and WB. F) Binding affinity of BtOBP2 with 1‐NPN. (G) Binding affinity of OBP2 to neophytadiene and other plant volatiles. ***P < 0.001. Bars in each graph depict mean ± SE; orange dots are replicates.

To identify which whitefly odorant‐binding proteins (OBPs) respond to neophytadiene, we characterized the relative gene expression levels of *OBP1‐8* following exposure to neophytadiene.^[^ ^31^, ^32^ ^]^ Neophytadiene was continuously released to whiteflies for 96 *h*, and *BtOBP1‐8* gene expression measured every 24 *h*. Only two genes, *BtOBP2* and *BtOBP3*, increased their expression over time, with *BtOBP2* exhibiting the most significant change (**Figure** **7**A).

The *BtOBP2*‐neophytadiene interaction was further explored by using RNA interference to silence the *BtOBP2* gene. After *BtOBP2* gene had been silenced, *BtOBP2* gene expression was suppressed (*p* < 0.001; Figure 7B), while other *OBPs* gene expression showed no difference (Figure S8, Supporting Information). ToCV accumulation in tomato plants after 30 d viral‐inoculation by viruliferous whiteflies was significantly reduced (*p* < 0.001; Figure 7C). These whiteflies exhibited no preference for neophytadiene in the Y‐tube olfactometer assay (*p* < 0.001; Figure 7D). However, after *BtOBP3* gene expression was suppressed as negative control (*p* < 0.001; Figure S9A, B, Supporting Information), whiteflies still exhibited preference for neophytadiene in the Y‐tube olfactometer assay (*p* < 0.001; Figure S9C, Supporting Information). These results suggest that expression of the *BtOBP2* olfactory protein in response to neophytadiene increases whitefly preference.

We next produced a purified recombinant BtOBP2 protein. A single band with a molecular weight (25‐35 *kDa*) was detected by sodium dodecyl sulfate polyacrylamide gel electrophoresis (SDS‐PAGE), consistent with the predicted molecular weight (27.33 *kDa*) (Figure S10A, Supporting Information). Western blot (WB) analysis confirmed the expression of the protein as BtOBP2 (Figure S10B, Supporting Information). Purification yielded a single band of the recombinant protein that was further confirmed by SDS‐PAGE and WB as BtOBP2 expression (Figure 7E). N‐Phenyl‐1‐naphthylamine (1‐NPN), a fluorescent probe commonly used to assess the affinity and specificity of BtOBPs to potential ligands,^[^ ^33^ ^]^ was employed to determine the binding affinity of BtOBP2 to 1‐NPN1. The binding of 1‐NPN showed a binding constant (*Kd*) of 2.94 ± 0.08 µM (Figure 7F). A competitive fluorescence binding assay was next used to determine the binding affinity of *BtOBP2* to neophytadiene and other plant volatiles detected in this research (Figure 7G). The median inhibitory concentration (IC_50_) and dissociation constant (*K_i_*) values were calculated,^[^ ^34^ ^]^ revealing that BtOBP2 exhibits a moderate binding affinity to neophytadiene, with an IC_50_ of 7.95 ± 0.45 µM and a *K_i_* of 2.02 ± 0.11 µM (**Table** **1**). These findings confirm that BtOBP2 has the strongest binding affinity for neophytadiene.

**Table 1:** Ligand binding affinities to BtOBP2.

Ligands	IC_50_ [µΜ]	*K_i_* [µΜ]
Neophytadiene	7.95 ± 0.45	2.02 ± 0.11
β‐pinene	13.67 ± 0.51	10.20 ± 0.38
β‐caryophyllene	14.25 ± 0.72	10.63 ± 0.54
α‐pinene	14.61 ± 0.57	10.90 ± 0.43
α‐terpinene	20.41 ± 1.29	15.23 ± 0.96
β‐phellandrene	25.41 ± 3.39	18.96 ± 2.53
ρ‐cymene	16.28 ± 0.49	12.15 ± 0.36
phytol	26.14 ± 1.48	19.51 ± 1.10

Affinities are given as means ± standard error of the mean (SEM). IC_50_, ligand concentration displacing 50% of the fluorescence intensity of the BtOBP2/1‐NPN complex; *K_i_*, dissociation constant.

Homology modeling and molecular docking predictions were analyzed to identify key binding sites in BtOBP2 for neophytadiene. According to the prediction with AlphaFold2, the Ramachandran diagram shows that 88.4% of the amino acid residues of the constructed protein structure were in the optimal region. The results of Ramachandran conformational maps of all predicted BtaOBP2 confirmed that the structure of BtaOBP2 satisfied the reasonability requirement, with the model of the amino acid residue percentage in the optimal region + allowable region + maximum allowable region greater than 90% (Figure S11, Supporting Information). The simulated structure of BtOBP2 consists of seven α‐helixes: α1 (residues Ser57‐Cys62), α2 (residues Glu91‐Lys106), α3 (residues Lys132‐Thr143), α4 (residues Glu154‐Lys166), α5 (residues Ala171‐Asn187), α6 (residues Ala198‐Ser217), and α7 (residues Glu222‐Lys233); and six disulfide bridges: Cys62‐Cys234, Cys79‐Cys224, Cys80‐Cys213, Cys99‐Cys138, Cys134‐Cys194, and Cys181‐Cys204 (Figure S12A, Supporting Information). Neophytadiene, a non‐polar compound, primarily interacted with the protein through hydrophobic forces. Phe210, Pro240, Pro74, Pro243 and Glu78 exhibited hydrophobic interactions with the small molecule at distances of 3.3 *A°*, 3.5 *A°*, 3.7 *A°*, 3.7 *A°*, and 3.1 *A°*, respectively (Figures S12B,C). The hydrophobic interaction between Phe210 and the small molecule was characterized by Pi‐Alkyl forces, while the others involved Alkyl hydrophobic interactions (Figure S12D, Supporting Information). These results confirmed the affinity of BtOBP2 for neophytadiene.

## 3 Discussion

**Figure 8:**
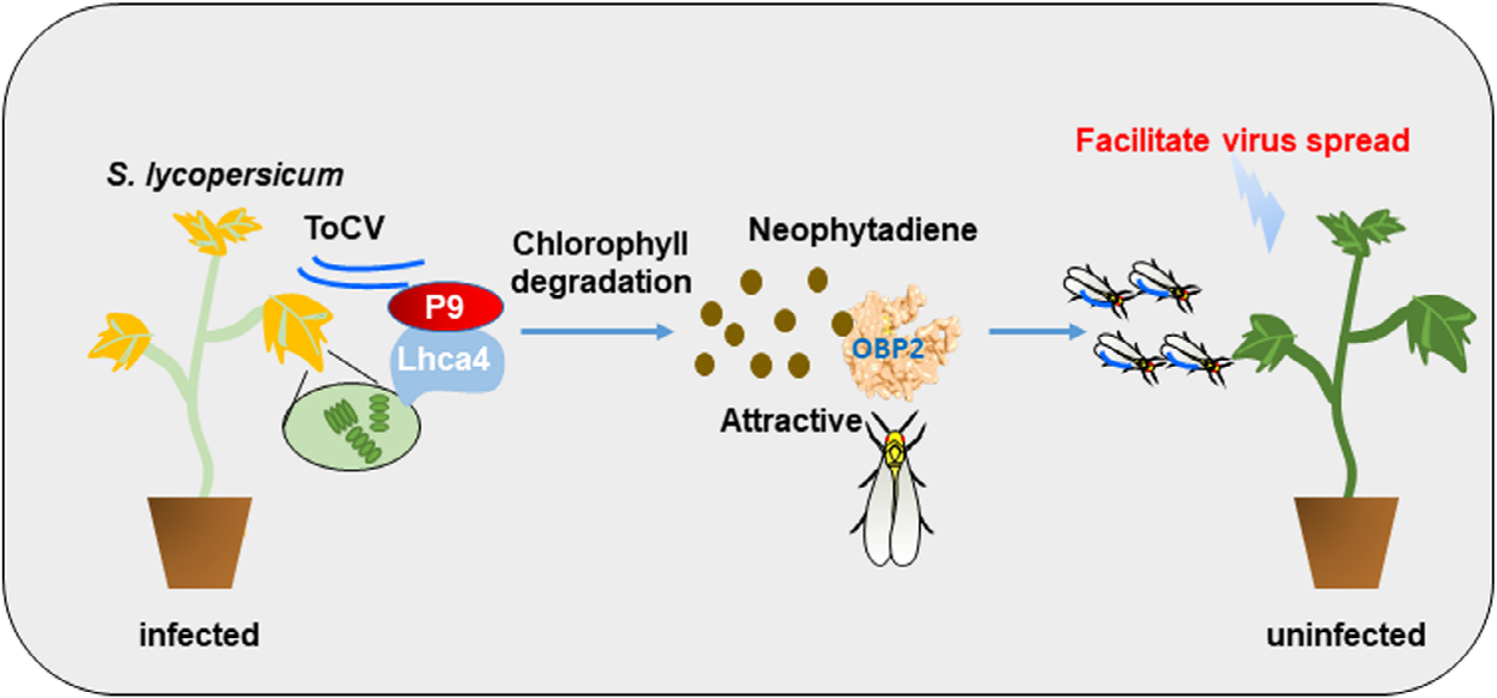
Pictorial representation of the mechanistic underpinnings of the virus‐vector‐whitefly interaction. ToCV infection induces the interaction of P9 and Lhca4, which promotes chlorophyll degradation to produce neophytadiene, and neophytadiene binds to OBP2, an odorant‐binding protein from *B. tabaci*.

Vector‐borne plant viruses have evolved an array of methods for manipulating plant and vector characteristics in ways that increase viral transmission.^[^ ^35^, ^36^, ^37^ ^]^ Our results revealed that ToCV manipulated both its tomato and tobacco host plants by triggering an increase in chlorophyll degradation and neophytadiene production that in turn increased vector preference and facilitated virus transmission. Interactions between the P9 ToCV protein and the Lhca4 plant protein increased chlorophyll degradation and neophytadiene production, which binds to the BtOBP2 whitefly protein and increased whitefly preference for infected plants (**Figure** **8**). Identifying the three key proteins involved in this virus‐plant‐vector interaction, and elucidating their functions, paves the way for developing more effective strategies to mitigate the impacts of viral diseases on crop productivity.

The genome of the *Crinivirus* tomato chlorosis virus (ToCV) comprises two RNA strands, RNA1 and RNA2,^[^ ^9^ ^]^ which encode 11 putative proteins: P4, P6, P7, P8, P9, P22, P27, P59, CP, CPm, and HSP70h. Among these, four ToCV proteins (CP, CPm, P9, and P22) were found to increase whitefly preference for infected host plants. The coat protein (CP) has been reported to enhance autophagy activity,^[^ ^17^ ^]^ influence the Ca^2+^‐dependent role in the oxidative stress response,^[^ ^38^ ^]^ and promote systemic viral infection.^[^ ^39^ ^]^ The minor coat protein (CPm) of cucurbit chlorotic yellows virus was recently found to interact with *B. tabaci* cytochrome c oxidase subunit 5A, tubulin beta chain, and type I cytoskeletal 9‐like keratin in ways that increase viral retention in the vector.^[^ ^40^ ^]^ The protein P22 interferes with SKP1‐Cullin‐F‐box^TIR1^ complex, attenuating auxin signaling and promoting ToCV infection.^[^ ^41^ ^]^ Notably, P9 is a putative 9 kDa protein encoded by RNA2, for which no specific function or similarity has been identified.^[^ ^9^ ^]^ Among these four proteins, P9 was the only one whose overexpression resulted in increased neophytadiene content; yeast two‐hybrid assays confirmed a direct interaction between P9 and Lhca4 (Figure 4). Plants overexpressing Lhca4 exhibited higher chlorophyll content and appeared greener, whereas ToCV infection and transient overexpression of P9 in Lhca4‐overexpressing plants produced contrasting symptoms (Figures 5 and 6).

Chlorophyll degradation is a critical biochemical process responsible for the yellowing associated with leaf senescence, and it has profound implications for intra‐plant nutrient recycling.^[^ ^42^ ^]^ Both biotic and abiotic factors regulate chlorophyll degradation, and viral infections often hasten this process.^[^ ^14^, ^43^ ^]^ In tomato plants, the Lhca4 protein is a part of the light‐harvesting complex (LHC) associated with photosystem I and is essential for efficient photosynthesis.^[^ ^44^ ^]^ This LHC protein thus plays a crucial role in capturing and transferring light energy to the reaction center of the photosystem. The P9 protein reduced Lhca4 expression, resulting in alterations to chloroplast structure and affecting the PSI‐LHCI protein, which leads to chlorophyll degradation and an accompanying increase in neophytadiene production in both tomato and tobacco plants.

The ability of neophytadiene to attract whiteflies has significant implications for our understanding of plant‐insect interactions. Odorant binding proteins (OBPs) are small soluble proteins found in the sensillar lymph of insect antennae and other olfactory organs. They bind and transport odor molecules through the aqueous sensillar lymph to the olfactory receptors, enabling insects to detect and respond to volatile signals.^[^ ^45^, ^46^ ^]^ Whitefly BtOBP2 reacted to neophytadiene but not to plant volatiles such as phytol and ρ‐cymene, suggesting that other OBPs are involved in identifying these and other compounds. *BtOBP2*‐silenced whiteflies showed no preference for ToCV‐infected plants and were less effective at transmitting ToCV to uninfected tomato plants, confirming that BtOBP2 affinity for neophytadiene enhances viral spread in this system. Despite the importance of whitefly attraction to neophytadiene, most research into virus‐vector‐plant interactions focusses on plant defense/deterrence toward natural enemies. The volatiles 1, 8‐cineole, p‐cymene, and limonene repelled the whitefly *Trialeurode vaporariorum* from settling on an otherwise‐preferred tomato cultivar,^[^ ^47^ ^]^ for example, while (Z)‐3‐hexenol improves tomato defense against infection by whitefly‐transmitted TYLCV.^[^ ^48^ ^]^ Further research into the role played by both attractive and repellent volatiles in determining vector preference may provide essential insights for improving control of insect‐mediated virus transmission in both wild and managed systems.

In conclusion, our findings provide key insights of genetic underpinnings governing the virus‐vector‐plant tripartite interactions, centering around a specialized metabolite in host plants. The critical roles played by ToCV P9, host plant Lhca4, whitefly *BtOBP2*, and neophytadiene in this intricate multitrophic dialogue suggest new avenues for managing this devastating virus in both wild and managed ecosystems.

## 4 Experimental Section

### Plant Materials, Whiteflies, and ToCV Inoculation

Tomato (*Solanum lycopersicum* Mill. cv. Micro‐Tom) and tobacco (*Nicotiana benthamiana*) were grown in insect‐free cages in a greenhouse (26 ± 2 °C, 60 ± 10% relative humidity, 16: 8 *L*: *D* photoperiod). *Bemisia tabaci* MED was cultured on tomato plants in metal‐nylon cages. The purity of the MED population was tested using PCR on the mitochondrial cytochrome oxidase I (*mtCOI*) gene (accession number: GQ371165) of 25 randomly selected whiteflies every other month. To achieve systemic infection, tobacco plants at the three‐true‐leaf stage were injected with an infectious ToCV cDNA clone.^[^ ^49^ ^]^ After visual confirmation of tobacco infection, whiteflies were used to transmit ToCV from tobacco to uninfected tomato plants. Tomato plants were inoculated by viruliferous whiteflies for 30 d before ToCV titres in the plants were measured using qRT‐PCR.^[^ ^49^ ^]^ Control plants were mock‐inoculated using whiteflies fed on uninfected tobacco plants grown under the same conditions. To generate Lhca4‐overexpressing tomato lines, the full‐length coding sequence of *Lhca4* was amplified by specific primers listed in Table S1 (Supporting Information) and cloned into the pBWA(V)HS‐osgfp vector. All vectors were transformed into *A. tumefaciens* strain GV3101 and introduced into tomato plants using the leaf disc method. The *KOLhca4*‐knockout mutants (*KOLhca4*) were produced using the CRISPR/Cas9 system. The sequence of three gRNA units was synthesized into the intermediate vector puc57 by whole genome synthesis, and then amplified by primers. Finally, the three gRNA units were ligated into the Cas9 expression vector (pHSbdcas9i). All binary vectors were transformed into *A. tumefaciens* strain GV3101 for transformation into “Micro‐Tom” tomato using the leaf‐disc method.^[^ ^50^ ^]^ Homozygous transgenic plants were used in all experiments. The designed guide RNA (gRNA, target 1/2/3) sequences and detection primers are listed in Table S1 (Supporting Information).

### Whole‐Plant Preference Tests

Tests were conducted as per Shi et al.^[^ ^49^ ^]^ with slight modifications. ToCV‐infected or control (mock‐inoculated) tomato plants were used for preference tests in screened cages. A second set of preference tests was also conducted using *OELhca4* (Lhca4 overexpressed) tomato plants sprayed either with neophytadiene (3 µg g^−1^ in water) or water only (control). Each cage contained two plants placed 40 cm apart. Under dim light, about 100 whiteflies that had been starved for 2 h were released above the center of the two plants and the whiteflies on each plant counted after 48 h. Each of the two preference tests (ToCV‐infected versus uninfected plants; *OELhca4* plants sprayed with either neophytadiene or water) was replicated nine times.

### Plant Volatile Assay and Y‐Tube Preference Tests

Volatile assays were conducted as per Shi et al.^[^ ^18^ ^]^ with slight modifications. Nine ToCV‐infected and nine control plants were used for volatile detection. Plant volatiles were collected for 6 h under continuous light, after which plants were weighed to determine volatile quantity expressed per g fresh weight (*FW*). Headspace samples were dissolved in n‐hexane and 0.2 µg mL^−1^ of n‐dodecane added to the solvent as an internal standard. Compounds were verified according to the National Institute of Standards and Technology (NIST) database.

Y‐tube preference assays were conducted as per Shi et al.^[^ ^51^ ^]^ with an air flow of 100 mL min^−1^. A volatile standard (Sigma Aldrich, USA) and control (solvent) were placed in glass containers at either end of the Y‐tube and 100 whiteflies were released at the Y‐tube base. The number of whiteflies moving more than 5 cm into the Y‐tube arm and remaining there for at least 15 s was counted for 20 min. There were nine replicates per volatile for each of the eight volatile compounds.

### Feeding Behavior

Whitefly feeding on ToCV‐infected or control tomato plants was recorded using the Electrical Penetration Graph (EPG) technique.^[^ ^52^, ^53^, ^54^ ^]^ EPG waveforms were recorded and observed in real time on a computer screen using a direct current eight‐channel Giga‐8d EPG device with Stylet+for Windows software (EPG stylet+d).^[^ ^55^ ^]^ EPG signals were recorded for 8 h inside a Faraday cage at 26 °C. Four waveforms were identified: *np* (non‐probing behavior), *C* (intercellular stylet pathway), *E1* (salivation into phloem sieve elements), and *E2* (phloem ingestion).^[^ ^56^ ^]^ Thirty biological replicates (= individual whiteflies) per treatment were analyzed.

### Plant Defense Response Detection

HPLC‐MS/MS was used to detect endogenous JA and SA. One g of fresh plant leaves was weighed and ground in liquid nitrogen. Crushed samples were added to 10 mL isopropanol/hydrochloric acid and shaken at 4 °C for 30 min; 20 mL dichloromethane was then added and shaken at 4 °C for 30 min before centrifugation at 13 000 rpm min^−1^ for 5 min at 4 °C. The organic phase was collected and dried with nitrogen in darkness. Hormones were dissolved with methanol (400 µL, 0.1% formic acid) and filtered through a filter membrane (0.22 µm). The HPLC reaction conditions were as follows: column (Waters ACQUITY UPLC R BEH C18, 100 mm × 2.1 mm × 1.7 µm), column temperature: 40 °C, mobile phase A: 98/2 = water/methanol (V/V)+0.05% formic acid +5 mmol L^−1^ ammonium acetate, mobile phase B: acetonitrile, flow rate: 0.3 mL min^−1^, injection volume: 5 µL, sample room temperature: 10 °C. The MS reaction conditions were as follows: ionization method: ESI positive and negative ion switch, capillary voltage: 3.0 kV, ion source temperature: 150 °C, cone hole blowback air flow: 50 L h^−1^, desolventizing gas temperature: 400 °C, desolventizing gas flow: 800 L h^−1^, and monitoring mode: MRM mode.

### Plasmid Construction

The ToCV virus genes CPm, CP, P4, P6, P7, P8, P9, P22, P59, and HSP70 h (Table S1, Supporting Information) were amplified and connected to the transient expression vector PVX pGR106 using the Phanta Max Super‐Fidelity DNA Polymerase kit (Vazyme, Nanjing China) and the ClonExpress II One Step Cloning Kit (Vazyme, Nanjing China). Following sequence verification the constructs were transformed into the *Agrobacterium tumefaciens* strain GV3101 and infiltrated into tobacco and tomato leaves. After 15 d, RT‐PCR was used on infiltrated leaves to confirm they contained the target genes.

### Chlorophyll Determination

OK‐Y104 chlorophyll meter (Oukeqi Manufacturing Co. Ltd., Zhengzhou China) was used to measure tomato leaf chlorophyll. Three technical replicates and three biological replicates per group (treatment, control) were analyzed.

### Yeast Two‐Hybrid Assay

BD‐ToCV P9 was used as the bait protein to screen the tomato yeast library. After the activated library liquid was spread on SD/−Leu/−Trp double‐dropout (DDO) medium, monoclonal yeast was picked and streaked on SD/‐Leu/‐Trp/‐His/‐Ade quadruple dropout (QDO) medium. ADT7 and AD3R primers were used to clone the gene after three days of inverted culture in an incubator at 30 °C; the gene sequence of the interaction protein was obtained by sequencing. Lhca4 and ToCV P9 were ligated to pGADT7 plasmid and pGBKT7 plasmid, respectively, and the yeast expression plasmids of pGADT7‐Lhca4 and pGBKT7‐P9 obtained. As per the yeast two‐hybrid method,^[^ ^41^ ^]^ the recombinant plasmids pGADT7‐Lhca4 and pGBKT7‐P9 were co‐transformed into yeast, plated on DDO medium and QDO medium respectively, and observed after three days of inverted culture in an incubator at 30 °C.

### Bimolecular Fluorescence Complementation (BiFC) Assay and Co‐Localization

For the BiFC assay, P9 and Lhca4 were connected to the N‐terminus and C‐terminus of the yellow fluorescent protein (YFP), respectively. The recombinant and empty vector were then transformed into *A. tumefaciens* GV3101. Cultured cells were harvested, resuspended in infiltration buffer, and infiltrated into tobacco leaves. For co‐localization analysis, the mixture of P9‐GFP and Lhca4‐mCherry was infiltrated into tobacco leaves. After 36 h, a confocal laser scanning microscope (Nikon C2 plus) was used to detect fluorescent signals of protein interaction. Primer information is listed in Table S1 (Supporting Information).

### Co‐Immunoprecipitation (Co‐IP) Assay

For Co‐IP analysis, the full‐length coding sequences of Lhca4 and P9 were cloned into 1300–35S‐3*FLAG and 1300‐GFP vectors to produce Lhca4‐FLAG and green fluorescent protein (GFP)‐P9 constructs, respectively. Co‐IP assay was performed as per Zhang et al.^[^ ^57^ ^]^ with slight modifications. Briefly, the Lhca4‐FLAG vector was co‐transformed with the GFP‐P9 or GFP‐empty vector (as a negative control) into tobacco leaves. After incubation, total protein was extracted from leaf tissue, resuspended in 500 µL of extraction buffer (Tris‐HCl 50 mM, pH 7.5; NaCl 150 mM; DTT 1 mM; PMSF 1 mM; EDTA 2 mM; 0.1% Triton X‐100; 1 x protease inhibitor cocktail), and mixed at 4 °C for 30 min. Protein samples (50 µL) were stored as the input group, while the remaining samples were incubated with anti‐FLAG agarose beads (Millipore) for 3 h at 4 °C. The immunoprecipitates were washed four times with Co‐IP washing buffer (Tris–HCl 50 mM, pH 7.5, NaCl 150 mM, EDTA 5 mM, and 0.1% Triton) and once with Tris–HCl (50 mM, pH 7.5). Proteins were eluted from the beads with SDS buffer and boiled for 10 min. The input and IP proteins were analyzed by immunoblot with anti‐FLAG (1:500; Invitrogen) or anti‐GFP (1:5000; Invitrogen) antibodies. Primer information is listed in Table S1 (Supporting Information).

### Dual Luciferase Assay

Full‐length Lhca4 and P9 coding sequences were cloned into 1300‐nLUC and 1300‐cLUC vectors. Agro‐infiltration of tobacco leaves was done using different combinations of agrobacterium solution.^[^ ^50^ ^]^ After three days, fluorescence was detected using a luciferase assay kit (Vazyme, Nanjing China). Primer information is listed in Table S1 (Supporting Information).

### RNA Extraction and Quantitative Reverse Transcription–PCR

RNA was extracted using trizol reagent (Invitrogen, Waltham USA) and dissolved in double‐deionized water; cDNA was synthesized from total RNA (1 µg) using HiScript 1st Strand cDNA Synthesis kit (Vazyme, Nanjing China). Real‐time qPCR was conducted using the MonAmp SYBR Green qPCR Mix (Thermofisher, Waltham USA). *Actin* and *UBI* were selected as the housekeeping genes (Table S1, Supporting Information). Each experiment was repeated at least three times and relative gene expression levels calculated for analysis using the 2^−∆∆Ct^ method.

### Proteomic Analysis

Total protein was extracted using a urea lysis buffer (8 M urea and 1% SDS) with a protease inhibitor. Protein concentrations were determined using a bicinchoninic acid (BCA) Protein Assay Kit (Thermofisher, Waltham USA). Then protein (100 µg) was taken and TEAB was added to make a final concentration of 100 mM. TCEP (tris (2‐carboxyethyl) phosphine) was then added to make a final concentration of TCEP 10 mM, and the reaction was carried out at 37 °C for 60 min. IAM (Iodoacetamide) was then added to make a final concentration of IAM 40 mM, and the reaction was conducted at room temperature for 40 min in the dark. Precooled acetone (acetone: sample volume = 6:1) was added to each tube, precipitated at −20 °C for 4 h, and centrifuged at 10 000×g for 20 min. The precipitate was fully dissolved with TEAB (100 µL of 100 mM), and trypsin at a mass ratio of 1:50 (enzyme: protein) was added for overnight enzymatic hydrolysis at 37 °C.

TMT reagent (No. A44522, Thermofisher, Waltham USA) was taken out at −20 °C and brought to room temperature. Acetonitrile was added and centrifuged by a vortex mixer. One tube of TMT reagent was added for every 100 µg of polypeptide and incubated at room temperature for 2 h. Hydroxylamine was then added and reacted at room temperature for 30 min. The labeled products were then mixed in a tube with the same amount and drained with a vacuum concentrator before all samples were pooled and vacuum dried.

High pH reverse phase separation was used to fractionate samples in order and increase proteomic depth. Peptide samples were re‐solubilized with UPLC loading buffer (2% acetonitrile (ammonia to pH 10)) and separated in high pH liquid phase using a reversed‐phase C18 column ACQUITY UPLC BEH C18 Column (1.7 µm, 2.1 mm × 150 mm, Waters Corp., Milford USA). 2D analysis was performed by LC‐ tandem MS (Easy‐nLC 1200 combined with Q Exactive HF‐X mass spectrometer) according to the standard protocols (Majorbio Bio‐Pharm Technology, Shanghai, China). The peptide mixture was loaded onto the C18 column (75 µm × 25 cm, Thermo, USA) for liquid phase separation in solvent A (2% ACN with 0.1% formic acid) and a linear gradient of solvent B (80% ACN with 0.1% formic acid) at a flow rate of 300 nL min^−1^.

The Q_Exactive HF‐X was operated in the data‐dependent acquisition mode (DDA) to automatically switch between full scan MS and MS/MS acquisition. The full scan MS spectra (m/z 350–1500) were acquired in the Orbitrap with 120 K resolution. Precursor ions were then selected into the collision cell for fragmentation by higher‐energy collision dissociation (HCD). The MS/MS resolution was set at 45 K, and dynamic exclusion was 20 s.

Raw data files were analyzed using Proteome Discoverer v2.4 (Thermofisher, Waltham USA) against the tomato database. Precursor mass tolerance was set at 20 ppm and fragment mass tolerance was set at 0.02 Da. The false discovery rate (FDR) of peptide identification was set as FDR ≤ 0.01. A minimum of one unique peptide identification was used to support protein identification.

Data were analyzed using the majorbio choud platform (cloud.majorbio.com). The thresholds of fold change (>1.2 or <0.83) and P‐value <0.05 were used to identify differentially expressed proteins (DEPs). Annotation of all identified proteins was performed using GO (http://geneontology.org/) and KEGG pathway (http://www.genome.jp/kegg/). DEPs were further used to for GO and KEGG enrichment analysis.

### Transmission Electron Microscopy Observation

Tomato leaves were cut into pieces and fixed overnight at 4 °C in fixation buffer (2.5% glutaraldehyde, phosphate 0.05 M, pH 7.2). Samples were then washed three times with fixation buffer followed by postfixation in 2% osmium tetroxide at 4 °C for 2 h. After dehydration in a graded ethanol series (50%, 70%, 80%, 90%, 95%, and 100%), the tissue samples were embedded in Spurr's resin. Ultrathin sections (70–90 nm) were cut using a Leica EM UC ultramicrotome and sequentially stained with uranyl acetate for 20 min and Reynolds' lead citrate for 5 min. The sections were viewed with a JEM‐1230 transmission electron microscope (JEOL, Tokyo Japan) operated at 80 kV.

### Separation of Thylakoid Membrane and Protein Analysis

Thylakoids were prepared as per Järvi et al.^[^ ^58^ ^]^ with slight modifications. Four types of plants were used: wild‐type plants, *OELhca4* plants, wild‐type plants transiently overexpressing the P9 protein, and *OELhca4* plants transiently overexpressing the P9 protein. Briefly, veins were removed from 1.5 g of plant leaves and the remaining tissue was cut into 1–3 cm^2^ fragments and immersed in 10 mL of pre‐cooled thylakoid membrane extraction buffer. The solution was then transferred into an electric homogenizer and homogenized at low speed for 5 s to avoid foaming. The homogenate was then filtered and divided into four pre‐cooled 15 mL plastic centrifuge tubes. These were centrifuged at 4 °C, 4200 g for 15 min, after which the supernatant was discarded from the thylakoid membrane precipitate.

The outer tank of BN‐PAGE electrophoresis used 1×BN/CN electrophoresis buffer. At the beginning of the inner tank electrophoresis, 1× blue cathode buffer was used. Fifteen *uL* samples were added to each hole with a regulated voltage 120 V (current 10 mA) for ice bath electrophoresis. When the electrophoretic indicating front reached one‐third of the way on the gel, the electrophoretic buffer was replaced with 1×BN/CN electrophoretic buffer, the voltage was adjusted to 120 V (current 4 mA) and electrophoresis continued until the indicated dye reached the bottom edge.

### Transcriptional Response of BtOBP1‐8 to Neophytadiene

To investigate the expression profiles of *BtOBP1‐8* genes in *B. tabaci* under neophytadiene treatment, the following experiment was performed. Tomato plants with an identical growth cycle were selected as the treatment group (sprayed with 3 µg g^−1^ neophytadiene) and the control group (treated with water only). The treated plants were then placed in a 10 L hermetic container. Once the volatilization reached equilibrium, 500 pairs of adults were introduced into the closed container and fumigated under airtight conditions. Subsequently, the whiteflies were sampled at distinct time points (0, 24, 48, 72, 96 h) respectively for the determination of expression levels.

### Bacterial Expression and Purification of BtOBP2

The specific primers for *BtOBP2* were designed using Primer 5 software to amplify the coding sequence according to the Phanta Max Super‐Fidelity DNA Polymerase protocol. Target DNA fragments were purified and ligated into the pEASY‐Blunt cloning vector, and positive colonies were identified by PCR and sequenced. The cloned plasmid was ligated into the pET28a vector (Invitrogen, Waltham USA) following digestion with BamHI and SalI. The resulting construct was transformed into Rosetta (DE3) *Escherichia coli* competent cells. When the OD600 of the recombinant BtOBP2 reached 0.6 to 0.8, induction was initiated by adding isopropyl‐β‐D‐1‐thiogalactopyranoside (IPTG) (1 mM) at 16 °C for 16 h, followed by purification using the Ni‐NTA purification system.^[^ ^59^ ^]^ The target protein was verified by 10% SDS‐PAGE, and its concentration was determined with a BCA protein assay kit. The recombinant purified protein was stored at −80 °C until use. The target protein was subjected to Coomassie blue staining and western blot validation through 10% SDS‐PAGE using anti His‐Tag antibody, and its concentration was determined using a BCA protein assay kit. The purified recombinant protein was stored at −80 °C.

### Fluorescence Competitive Binding Assays

The binding affinity of *BtOBP2* to the fluorescent probe N‐phenyl‐1‐naphthyl‐amine (1‐NPN1) was determined using a FL7000 fluorescence spectrophotometer (Hitachi, Tokyo Japan). Ligands and 1‐NPN were dissolved in HPLC‐grade methanol to a concentration of 1 mM, and then titrated with *BtOBP2* (2 µM) to final concentrations ranging from 2 to 20 µM.^[^ ^60^ ^]^ The optimal excitation and emission wavelengths of the recombinant protein *BtOBP2* were determined, and the 1‐NPN/*BtOBP2* mixture was excited at 220 nm with emission spectra recorded from 300 to 500 nm. The binding constants (*Kd*) of *BtOBP2* to 1‐NPN were calculated by Scatchard analysis using Prism 5 software. The IC_50_ represents the concentration at which 50% of the probe was displaced by the ligand. The K_i_ values were calculated according to the following equation: K_i_ = [IC_50_]/(1 + [1‐NPN]/K1‐NPN), where [1‐NPN] is the free concentration of 1‐NPN, and K1‐NPN is the Kd of the 1‐NPN/*BtOBP2* complex.^[^ ^61^ ^]^ The binding affinity between *BtOBP2* and ligands was classified based on their Ki values: values <20 µM indicated strong binding affinity, 20–50 µM values indicated moderate affinity, 50–100 µM values indicated weak affinity, and >100 µM values showed no affinity.^[^ ^34^ ^]^

### Homology Modeling and Molecular Docking

The protein sequence of BtOBP2 was retrieved from NCBI. To predict which amino acids were crucial for the interaction between BtOBP2 and neophytadiene, we first performed homology modeling of BtOBP2 using AlphaFold2 to construct the 3D model of protein. Ramachandran plots of each amino acid residues in the modeled proteins were plotted by SAVES server (https://SAVES.mbi.ucla.edu/) to evaluate the quality of the predicted model. The 2D structure of ligands was obtained from PubChem (https://pubchem.ncbi.nlm.nih.gov) and Chem3D was employed for structure optimization. Molecular docking was performed by AutoDock 4.2 software wherein the predicted structure of BtOBP2 was used as the receptor. The entire protein was wrapped in a docking box, and 200 conformations were searched. The lowest‐energy conformation was selected and visualized with pymol.^[^ ^62^ ^]^

### RNA Interference of BtOBP2 Gene and Virus Transmission Detection

The *BtOBP2* gene was amplified with healthy whitefly cDNA as a template using a specific primer containing the T7 promoter sequence. The GFP gene sequence was amplified by GFP PCR products and primers (Table S1, Supporting Information). The dsRNA synthesis conditions and RNA interference were as per Shi et al.^[^ ^18^ ^]^ After dsRNA treatment, *BtOBP2* expression in whiteflies was measured using RT‐qPCR. After being treated with *dsGFP* and *dsBtOBP2*, whitefly preference for neophytadiene was assessed in a preference experiment as per Shi et al.^[^ ^49^ ^]^ Each preference experiment was repeated nine times in separate cages. Viral transmission by whiteflies treated with *dsGFP* and *dsBtOBP2* was assessed as per Lu et al.^[^ ^63^ ^]^ with slight modifications. Each clip cage (50 whiteflies/cage) was attached to a ToCV‐infected tomato plant for a 48‐h virus acquisition period. Each whitefly‐containing clip cage was then transferred to a healthy tomato plant for a 48‐h viral inoculation period. Plants were then grown without further exposure to *B. tabaci* or ToCV for 30 d, after which ToCV accumulation was determined. The experiment was replicated six times.

### Statistical Analysis

SPSS 22.0 (SPSS Inc., Chicago, IL, USA) was used for statistical analysis. The general linear model (GLM) was used to compare whitefly preference and plant volatiles. Whitefly feeding behavior and endogenous phytohormone content were compared with independent‐sample *t*‐tests. One‐way ANOVA and Tukey's HSD post‐hoc test were used to compare the differences in relative gene expression on healthy plants and ToCV‐infected plants, and neophytadiene content on tobacco plants with ToCV proteins overexpressed. Independent‐sample *t*‐tests were used to compare chlorophyll content, relative gene expression, number of whiteflies, and neophytadiene content on wild type and *Lhca4* overexpressed tomato plants (with or without virus, with or without P9 protein). The general linear model (GLM) was used to compare whitefly preference, neophytadiene content, and chlorophyll content on wild type and *KOLhca4* plants with or without virus. One‐way ANOVA and Tukey's HSD post‐hoc test were used to compare *BtOBPs* gene expression from 0 h to 96 h. Independent‐sample t‐tests were used to compare *BtOBP2* gene expression and ToCV accumulation after whiteflies feeding with *dsGFP* and *dsBtOBP2*. Two‐way ANOVAs were used to compare whitefly preference to control and neophytadiene after whiteflies were fed with either *dsGFP* or *dsBtOBP2*.

## Conflict of Interest

The authors declare no conflict of interest.

## Author Contributions

X.B.S., H.Y., and Y.W. contributed equally to this work. X.S., X.Z., and Y.L. designed and supervised the projects. H.Y., Y.W., Z.Z., J.D., K.L., and J.C. performed the experiments, drafted the manuscript, and created the figures. X.S., H.Y., E.P., and Y.W. analyzed data. X.S. H.Y., E.P., and Y.W. wrote the paper. X.Y., S.Z., Z.Z., and D.Z. provided technical support and revised the manuscript. All the authors have read and approved the final version of the manuscript. All the listed authors have read and approved the final version, including all details and images.

## Supporting information



Supporting Information

Supporting Information

## Data Availability

The data that support the findings of this study are available from the corresponding author upon reasonable request.

## References

[1] S. Crawshaw , L. G. Watt , A. M. Murphy , J. P. Carr , J. Virol. 2024, 98, 0099324. 10.1128/jvi.00993-24 PMC11406993 39162432

[2] S. Chen , X. Zhong , J. Integr. Plant Biol. 2024, 66, 2000. 38923382 10.1111/jipb.13722

[3] L. Zhang , Y. Li , J. H. Kuhn , K. Zhang , Q. Song , F. Liu , PLoS Pathog. 2024, 20, 1012112. 10.1371/journal.ppat.1012112 PMC10984529 38507423

[4] S. Tian , Q. Song , W. Zhou , J. Wang , Y. Wang , W. An , Y. Wu , L. Zhao , Mol. Plant 2024, 17, 614. 38454602 10.1016/j.molp.2024.03.004

[5] M. Jeger , F. Hamelin , N. Cunniffe , Phytopathology 2023, 113, 1630. 36647183 10.1094/PHYTO-10-22-0378-V

[6] K. Mauck , Q. Chesnais , Virus Res. 2020, 285, 197957. 32380208 10.1016/j.virusres.2020.197957

[7] W. M. Wintermantel , G. C. Wisler , A. G. Anchieta , H. Y. Liu , A. V. Karasev , I. E. Tzanetakis , Arch. Virol. 2005, 150, 2287. 16003497 10.1007/s00705-005-0571-4

[8] E. Fiallo‐Olive , J. Navas‐Castillo , Ann. Appl. Biol. 2022, 182, 29.

[9] E. Fiallo‐Olive , J. Navas‐Castillo , Mol. Plant Pathol. 2019, 20, 1307. 31267719 10.1111/mpp.12847 PMC6715620

[10] C. Gong , Z. Guo , Y. Hu , Z. Yang , J. Xia , X. Yang , W. Xie , S. Wang , Q. Wu , W. Ye , X. Zhou , T. C. J. Turlings , Y. Zhang , Adv. Sci. 2024, 11, 2 306 653. 10.1002/advs.202306653 PMC10933598 38145364

[11] K. K. Wei , J. Li , T. B. Ding , T. X. Liu , D. Chu , J. Integr. Agric. 2019, 18, 2107.

[12] L. Medina‐Puche , H. Tan , V. Dogra , M. Wu , T. Rosas‐Diaz , L. Wang , X. Ding , D. Zhang , X. Fu , C. Kim , R. Lozano‐Duran , Cell 2020, 182, 1109. 32841601 10.1016/j.cell.2020.07.020

[13] Z. Shen , X. Yang , Y. Sun , C. Jiang , L. Cheng , D. Liu , L. Wen , A. Yang , Int. J. Biol. Macromol. 2024, 262, 130100. 38350582 10.1016/j.ijbiomac.2024.130100

[14] D. Bhattacharyya , S. Chakraborty , Mol. Plant Pathol. 2018, 19, 504. 28056496 10.1111/mpp.12533 PMC6638057

[15] M. Zhang , Y. Rao , X. Chen , Y. Shi , C. Wei , X. Wang , L. Wang , C. Xie , C. Pan , J. Chen , Front. Plant Sci. 2023, 14, 1 245 555. 10.3389/fpls.2023.1245555 PMC10579580 37854114

[16] S. Zhu , S. Sun , W. Zhao , X. Yang , H. Mao , L. Sheng , Z. Chen , BMC Plant Biol 2024, 24, 360. 38698342 10.1186/s12870-024-05076-7 PMC11067083

[17] J. B. Luan , D. M. Yao , T. Zhang , L. L. Walling , M. Yang , Y. J. Wang , S. S. Liu , Ecol. Lett. 2013, 16, 390. 23279824 10.1111/ele.12055

[18] X. Shi , E. L. Preisser , B. Liu , H. Pan , M. Xiang , W. Xie , S. Wang , Q. Wu , C. Li , Y. Liu , X. Zhou , Y. Zhang , BMC Plant Biol 2019, 19, 556. 31842757 10.1186/s12870-019-2178-z PMC6916021

[19] X. Shi , H. Pan , H. Zhang , X. Jiao , W. Xie , Q. Wu , S. Wang , Y. Fang , G. Chen , X. Zhou , Y. Zhang , Sci. Rep. 2014, 4, 5230. 24912756 10.1038/srep05230 PMC4050386

[20] X. Shi , H. Pan , W. Xie , Q. Wu , S. Wang , Y. Liu , Y. Fang , G. Chen , X. Gao , Y. Zhang , PLoS One 2013, 8, 83520. 10.1371/journal.pone.0083520 PMC3877053 24391779

[21] Y. Zheng , Y. Feng , Z. Li , J. Wang , Arch. Insect Biochem. Physiol. 2024, 115, 22102. 10.1002/arch.22102 38500452

[22] D. Wu , T. Qi , W. X. Li , H. Tian , H. Gao , J. Wang , J. Ge , R. Yao , C. Ren , X. B. Wang , Y. Liu , L. Kang , S. W. Ding , D. Xie , Cell Res. 2017, 27, 402. 28059067 10.1038/cr.2017.2 PMC5339842

[23] C. L. Casteel , M. De Alwis , A. Bak , H. Dong , S. A. Whitham , G. Jander , Plant Physiol. 2015, 169, 209. 26091820 10.1104/pp.15.00332 PMC4577379

[24] P. Li , C. Liu , W. H. Deng , D. M. Yao , L. L. Pan , Y. Q. Li , Y. Q. Liu , Y. Liang , X. P. Zhou , X. W. Wang , PLoS Pathog. 2019, 15, 1007607. 10.1371/journal.ppat.1007607 PMC6400417 30789967

[25] X. Wu , S. Xu , P. Zhao , X. Zhang , X. Yao , Y. Sun , R. Fang , J. Ye , PLoS Pathog. 2019, 15, 1007897. 10.1371/journal.ppat.1007897 PMC6598649 31206553

[26] H. Zhan , Y. Dewer , J. Zhang , J. Tian , D. Li , C. Qu , Z. Yang , F. Li , C. Luo , J. Agric. Food Chem. 2021, 69, 12785 785. 34669397 10.1021/acs.jafc.1c03825

[27] H. He , J. Li , Z. Zhang , M. Yan , B. Zhang , C. Zhu , W. Yan , B. Shi , Y. Wang , C. Zhao , F. Yan , Pest Manag. Sci. 2023, 79, 1410. 36480018 10.1002/ps.7313

[28] E. Elias , K. Brache , J. Schafers , R. Croce , J. Am. Chem. Soc. 2024, 146, 3508. 38286009 10.1021/jacs.3c13373 PMC10859958

[29] V. Yadavalli , S. Neelam , A. S. Rao , A. R. Reddy , R. Subramanyam , J. Plant Physiol. 2012, 169, 753. 22445751 10.1016/j.jplph.2012.02.008

[30] Z. A. Kiss , V. Medina , B. W. Falk , Front. Microbiol. 2013, 4, 99. 23730299 10.3389/fmicb.2013.00099 PMC3657685

[31] Y. Zeng , Y. T. Yang , Q. J. Wu , S. L. Wang , W. Xie , Y. Zhang , Insect Sci 2019, 26, 620. 29441682 10.1111/1744-7917.12576 PMC7380034

[32] J. Zhu , F. Wang , Y. Zhang , Y. Yang , D. Hua , J. Insect Sci. 2023, 23, 7. 10.1093/jisesa/iead004 PMC9894006 36729094

[33] F. Wu , C. Ma , B. Han , L. Meng , H. Hu , Y. Fang , M. Feng , X. Zhang , O. Rueppell , J. Li , Mol. Ecol. 2019, 28, 4212. 31444931 10.1111/mec.15207

[34] H. Li , Y. Chen , C. Lu , H. Tian , S. Lin , L. Wang , T. Linghu , X. Zheng , H. Wei , X. Fan , Y. Chen , PLoS Pathog. 2023, 19, 1011380. 10.1371/journal.ppat.1011380 PMC10194981 37155712

[35] K. E. Mauck , Curr. Opin. Virol. 2016, 21, 114. 27644035 10.1016/j.coviro.2016.09.002

[36] A. Biere , A. J. M. Tack , A. Bennett , Funct. Ecol 2013, 27, 646.

[37] E. K. Falla , N. J. Cunniffe , PLoS Comput. Biol. 2024, 20, 1012479. 10.1371/journal.pcbi.1012479 PMC11469505 39352908

[38] C. Liu , J. Zhang , J. Wang , W. Liu , K. Wang , X. Chen , Y. Wen , S. Tian , Y. Pu , G. Fan , X. Ma , X. Sun , Plant J. 2022, 112, 677. 36087000 10.1111/tpj.15972

[39] Y. J. Shi , X. Yang , L. L. Yang , Q. L. Li , X. M. Liu , X. Y. Han , Q. S. Gu , H. L. Li , L. L. Chen , Y. Liu , Y. Shi , Mol. Plant Pathol. 2023, 24, 208. 36528386 10.1111/mpp.13287 PMC9923391

[40] H. F. He , C. C. Zhao , C. Q. Zhu , W. L. Yan , M. H. Yan , Z. L. Zhang , J. L. Liu , B. Z. Shi , R. E. Bai , J. J. Li , F. M. Yan , Int. J. Biol. Macromol 2023, 226, 1154. 36427615 10.1016/j.ijbiomac.2022.11.229

[41] S. Liu , C. Wang , X. Liu , J. Navas‐Castillo , L. Zang , Z. Fan , X. Zhu , T. Zhou , Plant Cell Environ 2021, 44, 3155. 34105183 10.1111/pce.14125

[42] M. Yang , S. Zhu , B. Jiao , M. Duan , Q. Meng , N. Ma , W. Lv , Plant Physiol. Bioch. 2020, 157, 316. 10.1016/j.plaphy.2020.10.028 33166770

[43] J. Zhao , X. Zhang , Y. Hong , Y. Liu , Front. Microbiol. 2016, 7, 1565. 27757106 10.3389/fmicb.2016.01565 PMC5047884

[44] S. Jansson , Trends Plant Sci. 1999, 4, 236. 10366881 10.1016/s1360-1385(99)01419-3

[45] K. Rihani , J. F. Ferveur , L. Briand , Biomolecules 2021, 11, 509. 33808208 10.3390/biom11040509 PMC8067015

[46] J. S. Sun , S. Xiao , J. R. Carlson , Open Biol 2018, 8, 180 208. 10.1098/rsob.180208 PMC6303780 30977439

[47] E. Deletre , F. K. Matu , L. K. Murungi , S. Mohamed , J. Econ. Entomol. 2022, 115, 565. 35244166 10.1093/jee/toac015

[48] F. Yang , X. Zhang , H. Xue , T. Tian , H. Tong , J. Hu , R. Zhang , J. Tang , Q. Su , Plant J. 2022, 112, 694. 36086899 10.1111/tpj.15973

[49] X. Shi , X. Tang , X. Zhang , D. Zhang , F. Li , F. Yan , Y. Zhang , X. Zhou , Y. Liu , Front. Plant Sci. 2018, 8, 2271. 29387077 10.3389/fpls.2017.02271 PMC5776130

[50] H. J. Sun , S. Uchii , S. Watanabe , H. Ezura , Plant Cell Physiol. 2006, 47, 426. 16381658 10.1093/pcp/pci251

[51] X. Shi , G. Chen , H. Pan , W. Xie , Q. Wu , S. Wang , Y. Liu , X. Zhou , Y. J. Zhang , Front. Microbiol. 2018, 9, 1404. 29997607 10.3389/fmicb.2018.01404 PMC6030610

[52] B. Liu , E. L. Preisser , X. Shi , H. Wu , C. Li , W. Xie , S. Wang , Q. Wu , Y. Zhang , A. Biere , Funct. Ecol. 2017, 31, 1574.

[53] B. M. Liu , E. L. Preisser , X. Jiao , H. P. Pan , W. Xie , S. L. Wang , Q. J. Wu , Y. J. Zhang , Environ. Entomol. 2013, 42, 980. 24073848 10.1603/EN13071

[54] B. M. Liu , E. L. Preisser , D. Chu , H. P. Pan , W. Xie , S. L. Wang , Q. J. Wu , X. G. Zhou , Y. J. Zhang , J. Virol. 2013, 87, 4929. 23408638 10.1128/JVI.03571-12 PMC3624301

[55] N. K. P. Maluta , J. R. S. Lopes , E. Fiallo‐Olive , J. Navas‐Castillo , A. L. Lourencao , Insects 2020, 11, 559. 32842573 10.3390/insects11090559 PMC7565682

[56] N. Maluta , A. Fereres , J. R. S. Lopes , J. Pest Sci. 2018, 92, 405.

[57] H. Zhang , Z. Hu , C. Lei , C. Zheng , J. Wang , S. Shao , X. Li , X. Xia , X. Cai , J. Zhou , Y. Zhou , J. Yu , C. H. Foyer , K. Shi , Plant Cell 2018, 30, 652. 29511053 10.1105/tpc.17.00537 PMC5894845

[58] S. Järvi , M. Suorsa , V. Paakkarinen , E. M. Aro , Biochem. J. 2011, 439, 207. 21707535 10.1042/BJ20102155

[59] M. Brule , M. Glaz , C. Belloir , N. Poirier , L. Moitrier , F. Neiers , L. Briand , Methods Enzymol 2020, 642, 125. 32828250 10.1016/bs.mie.2020.05.002

[60] L. L. Li , J. R. Huang , J. W. Xu , W. C. Yao , H. H. Yang , L. Shao , H. R. Zhang , Y. Dewer , X. Y. Zhu , Y. N. Zhang , Pest Manag. Sci 2022, 78, 52. 34418275 10.1002/ps.6606

[61] C. Qu , Z. K. Yang , S. Wang , H. P. Zhao , F. Q. Li , X. L. Yang , C. Luo , Front. Physiol. 2022, 13, 829 766. 10.3389/fphys.2022.829766 PMC8957989 35350682

[62] S. Moradi , S. Khani , M. Ansari , M. Shahlaei , J. Mol. Liq. 2019, 276, 59.

[63] D. Lu , H. Yue , L. Huang , D. Zhang , Z. Zhang , Z. Zhang , Y. Zhang , F. Li , F. Yan , X. Zhou , X. Shi , Y. Liu , Pest Manag. Sci 2021, 77, 5294. 34310017 10.1002/ps.6572

