# Supplementary material for: Neophytadiene, a Plant Specialized Metabolite, Mediates the Virus‐Vector‐Plant Tripartite Interactions
Source: Adv Sci (Weinh). 2025 Apr 3;12(22):2416891. doi: 10.1002/advs.202416891 (PMC12165043; doi:10.1002/advs.202416891)
Supplement: Supplementary file 1 — Supporting Information [file ADVS-12-2416891-s001.docx]

Supporting Information

**Neophytadiene, a plant specialized metabolite, mediates the virus-vector-plant tripartite interactions**

*Xiao-bin Shi, Hao Yue, Yan Wei, Evan L. Preisser, Pei Wang, Jiao Du, Ji-xing Xia, Kai-long Li, Jian-bin Chen, Song-bai Zhang, Zhan-hong Zhang, Xu-guo Zhou, ^*^ De-yong Zhang, ^*^ and Yong Liu^*^*

**Figures S1-11, Table S1-2**

| **Figures and Tables** | **Content** | **Page** |
| --- | --- | --- |
| Figures S1 | Chromatographic peak of neophytadiene | 3 |
| Figures S2 | Process of neophytadiene release mediated by chlorophyll degradation | 4 |
| Figures S3 | Screening of proteins interacting with P9 by Yeast two-hybrid | 5 |
| Figures S4 | Location of P9-GFP and Lhca4-RFP in *N. benthamiana* leaf cells | 6 |
| Figures S5 | Plant height of WT and *Lhca4* over-expressed tomato plants (with or without ToCV infected) | 7 |
| Figures S6 | Phenotypes of Lhca4 knockout plants (*KOLhca4*) | 8 |
| Figures S7 | Comparative proteomics of wild type plants and wild plants with P9 over-expression | 9 |
| Figures S8 | Relative expression of *BtOBP1-8* in control (*dsGFP*) and *BtOBP2*-silenced (*dsOBP2*) whiteflies | 10 |
| Figures S9 | Whitefly preference to neophytadiene after *BtOBP3* was silenced | 11 |
| Figures S10 | Expression of the protein BtOBP2 | 12 |
| Figures S11 | Ramachandran plot of BtOBP2 produced by PROCHECK after homology modeling | 13 |
| Figure S12 | Homology modeling and molecular docking analysis for BtOBP2 | 14 |
| Table S1 | The main primers and probes used in this study | 15 |
| Table S2 | Differentially expressed proteins on WT and OELhca4 plants with or without P9 | 19 |


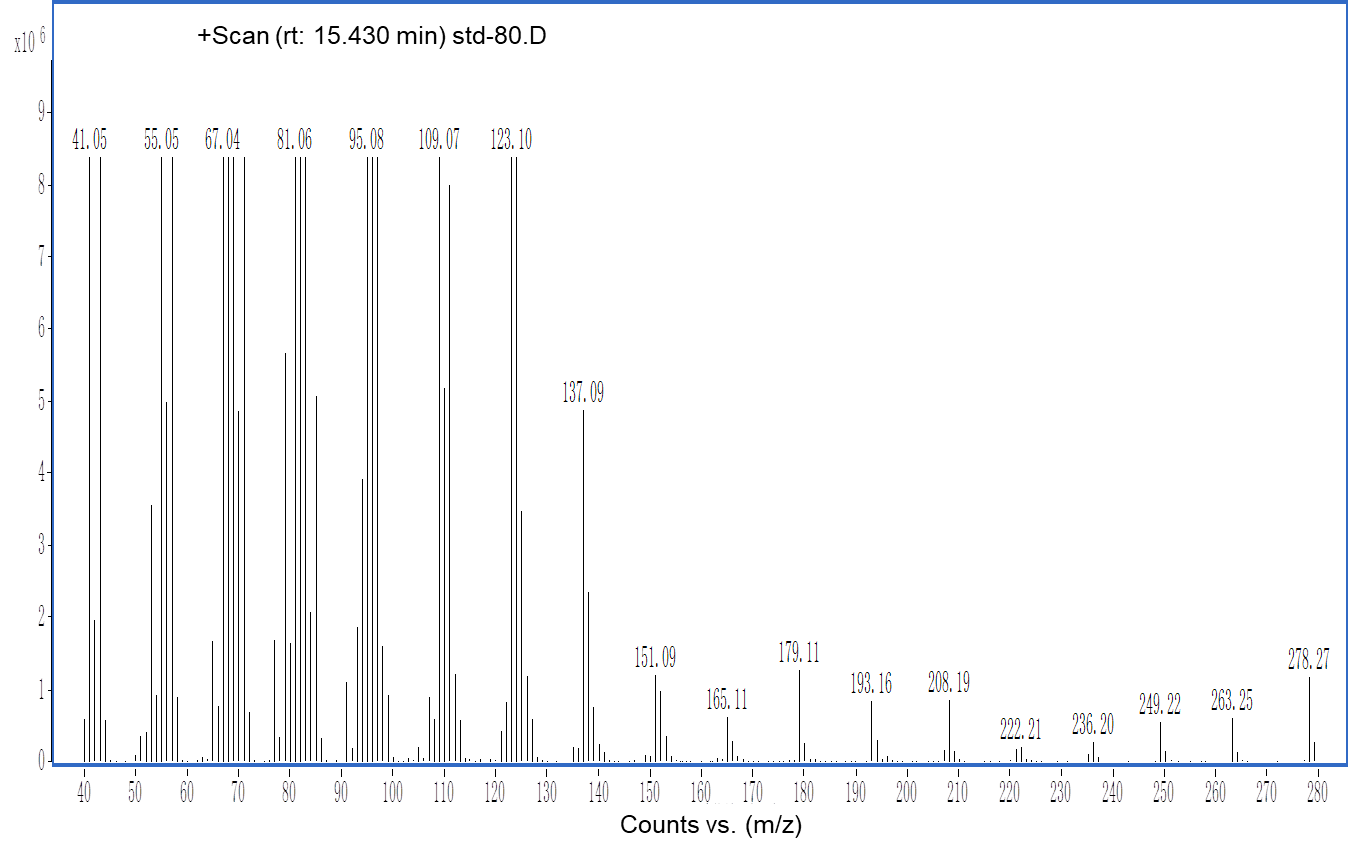


Figure S1. Chromatographic peak of neophytadiene.


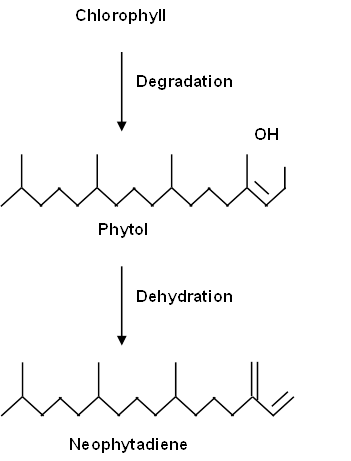


**Figure S2. Process of neophytadiene release mediated by chlorophyll degradation.**

**
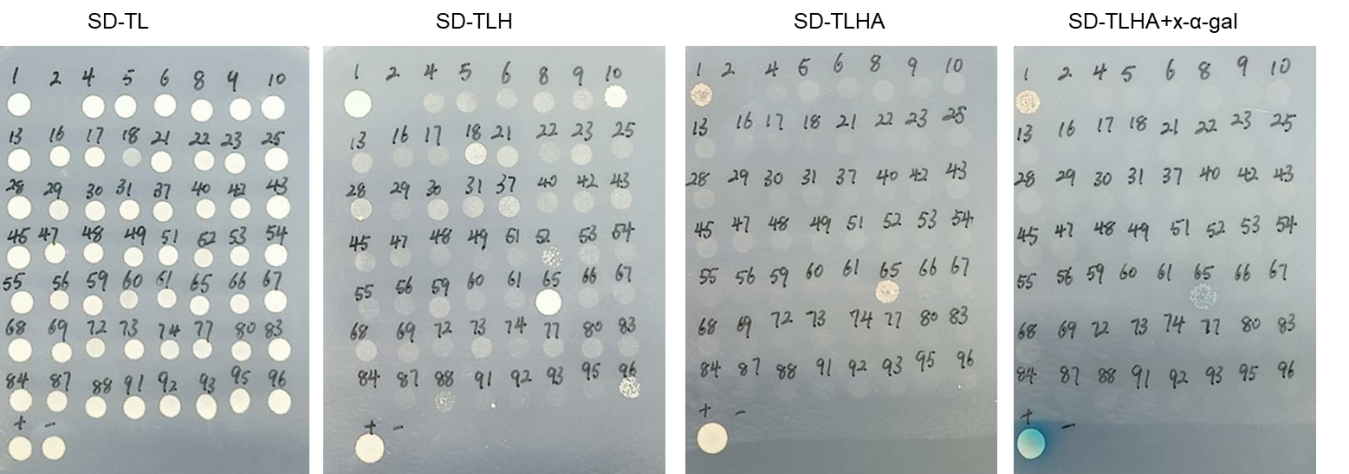
**

**Figure S3. Screening of proteins interacting with P9 by Yeast two-hybrid.** Yeast grows on a medium that lacks amino acids. T, Tryptophan; L, Leucine; H, Histidine; A, Adenine. SD-TL/TLH/TLHA represented the medium lacking corresponding amino acids, respectively.

**
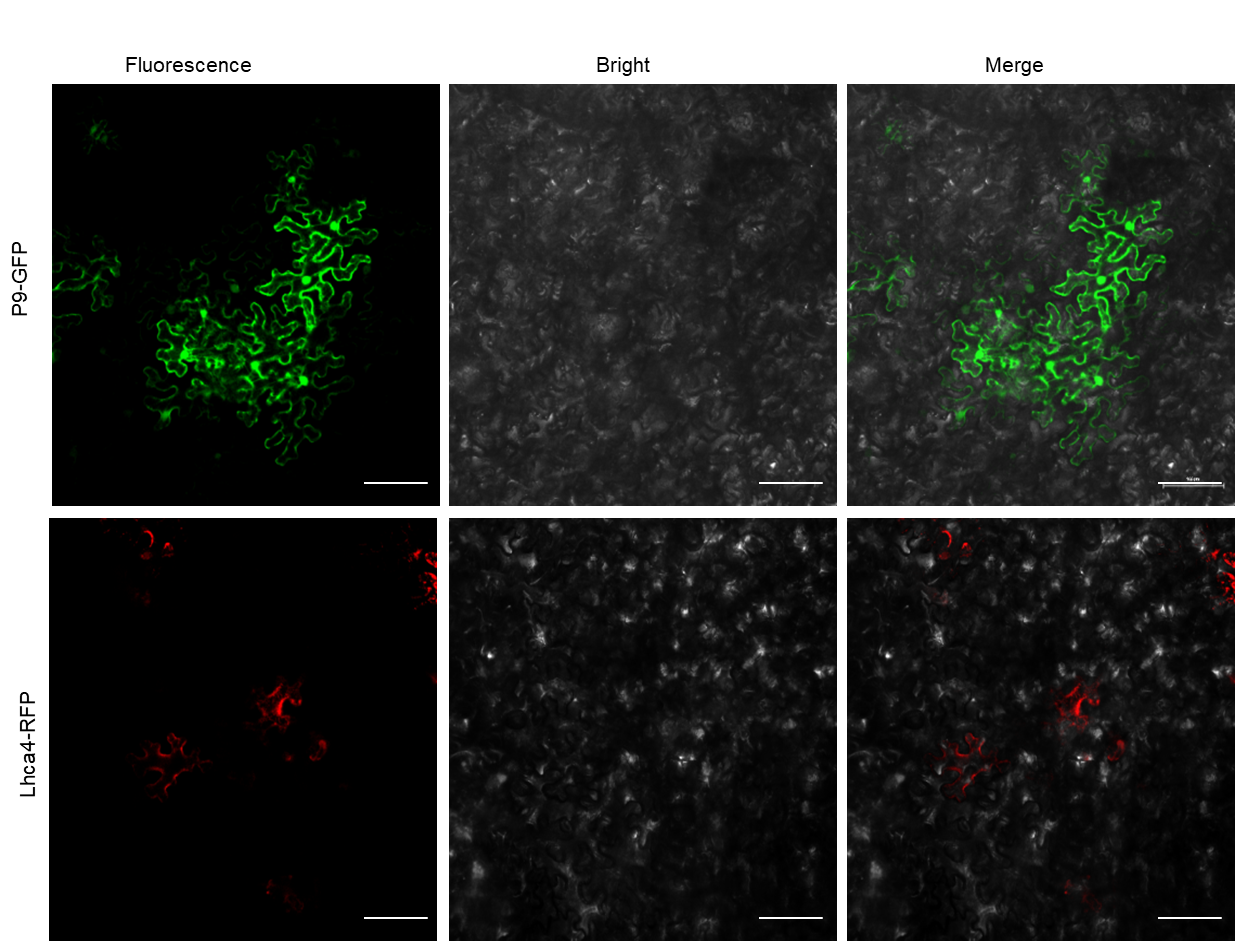
**

**Figure S4. Location of P9-GFP and Lhca4-RFP in** ***N. benthamiana* leaf cells.** GFP, ggreen fluorescent protein; RFP, red fluorescent protein; Bright, tthe brightness or intensity of a fluorescent signal; Merge, fluorescence signals from different channels are combined into one image.

**
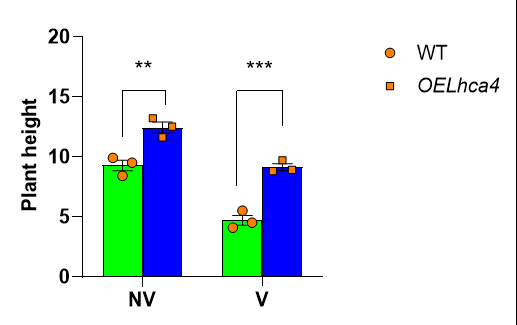
**

**Figure S5. Plant height of WT and *Lhca4* over-expressed tomato plants (with or without ToCV infected).** NV: Non-viruliferous tomato plants; V: viruliferous tomato plants. WT: wild type plants with green bars; OELhca4: OELhca4 tomato plants with blue bars.

**
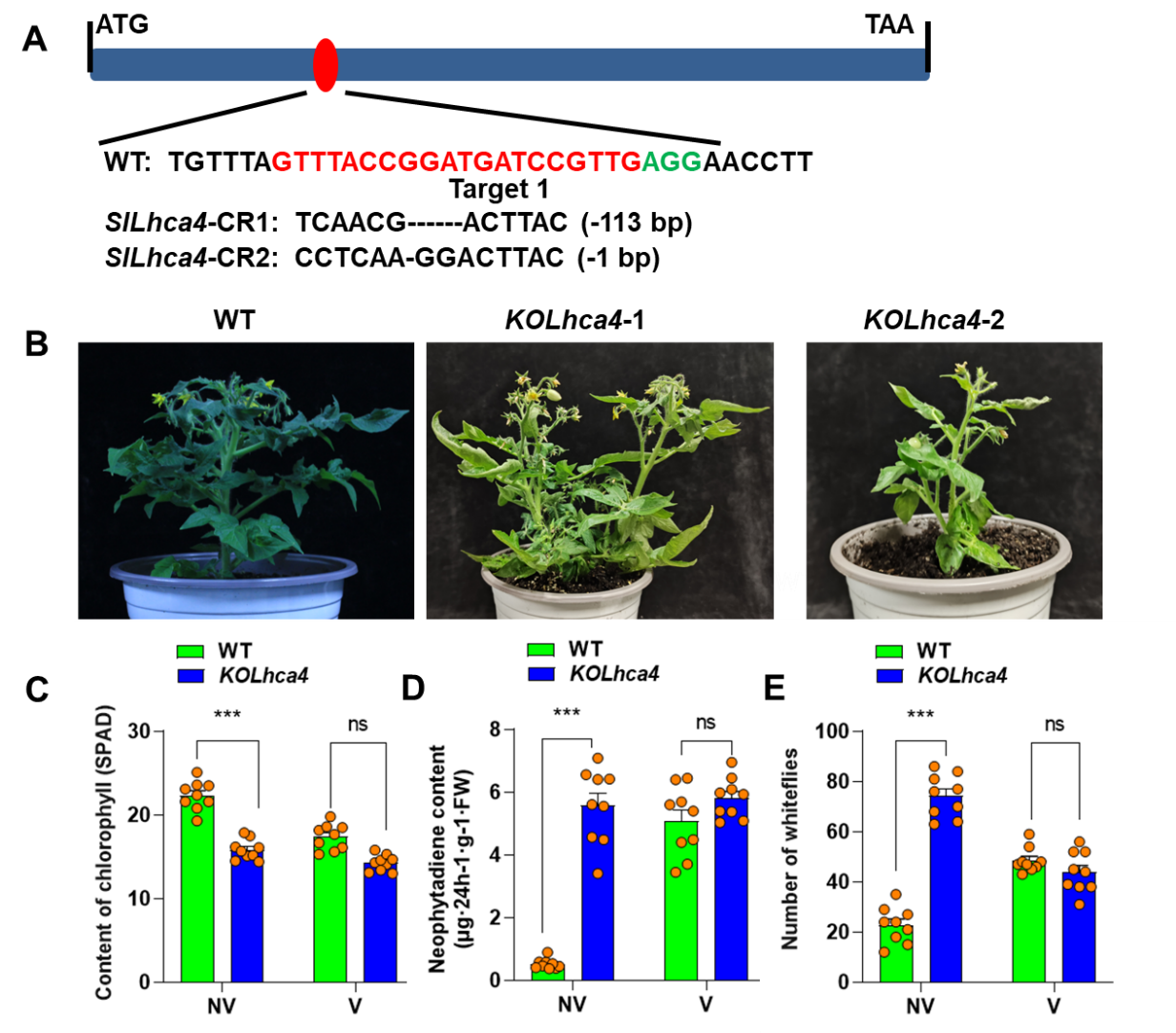
**

**Figure S6. Phenotypes of *Lhca4* knockout plants (*KOLhca4*).** (A) Schematic diagram of *KOLhca4* gene structure and CRISPR/Cas9-induced mutations at the target site. Blue box represents the exon of *KOLhca4*, and the red box indicates the position of designed gRNA. For sequences around gRNA in WT, the red letters represent gRNA sequence and the green letters represent PAM sequence. For mutations in *KOLhca4* –knockout plant (*KOLhca4-*1 and *KOLhca4-*2), the dashed line ‘-’ indicates a base deletion. (B) Plant phenotype of wild type and *KOLhca4* tomato. (C) Chlorophyll content of wild type and *KOLhca4* tomato. (D) Neophytadiene content of wild type and *KOLhca4* tomato. (E) Number of whiteflies on wild type and *KOLhca4* tomato. NV: non-viruliferous. V: viruliferous. ***P < 0.001. Bars in each graph depict mean + SE; orange dots are replicates.


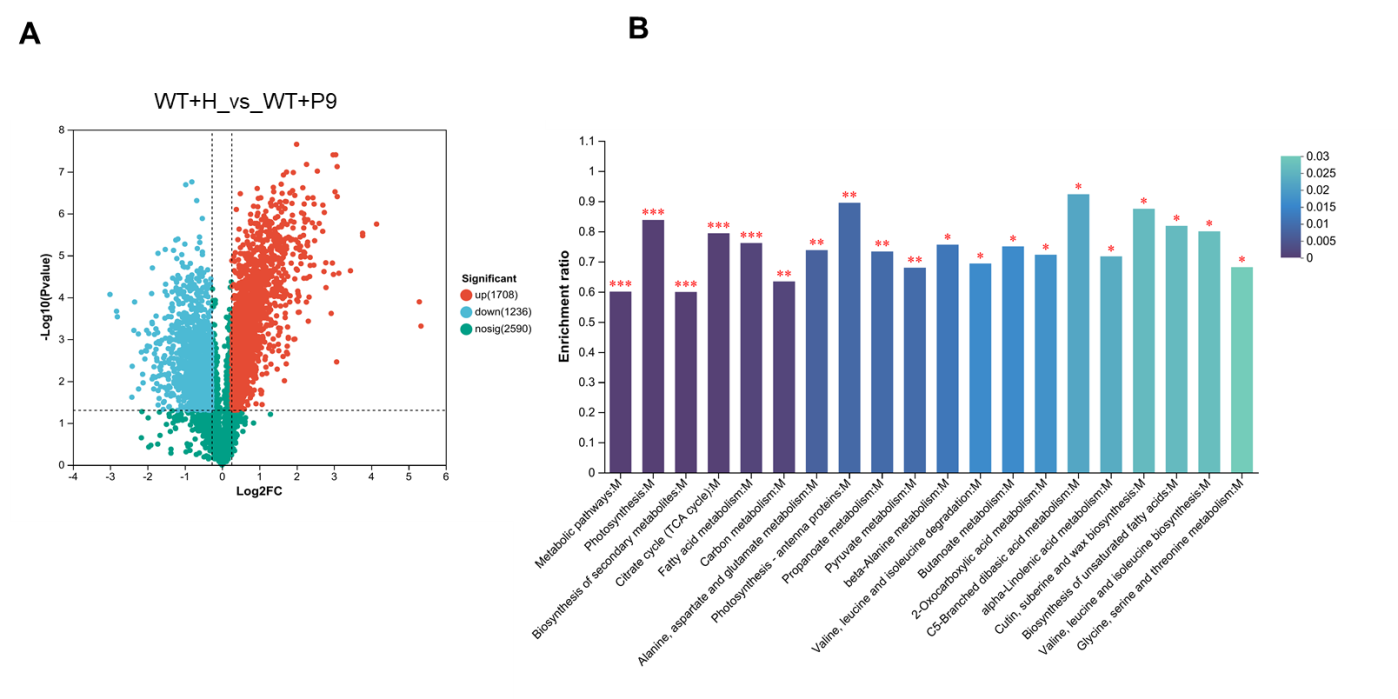


**Figure S7. Comparative proteomics of wild type plants and wild plants with P9 over-expression.** (A) Up- and down-regulated differentially expressed proteins in tomato plants (with or without P9 expression). (B) KEGG enrichment analysis of the differentially expressed proteins in tomato plants (with or without P9 expression).


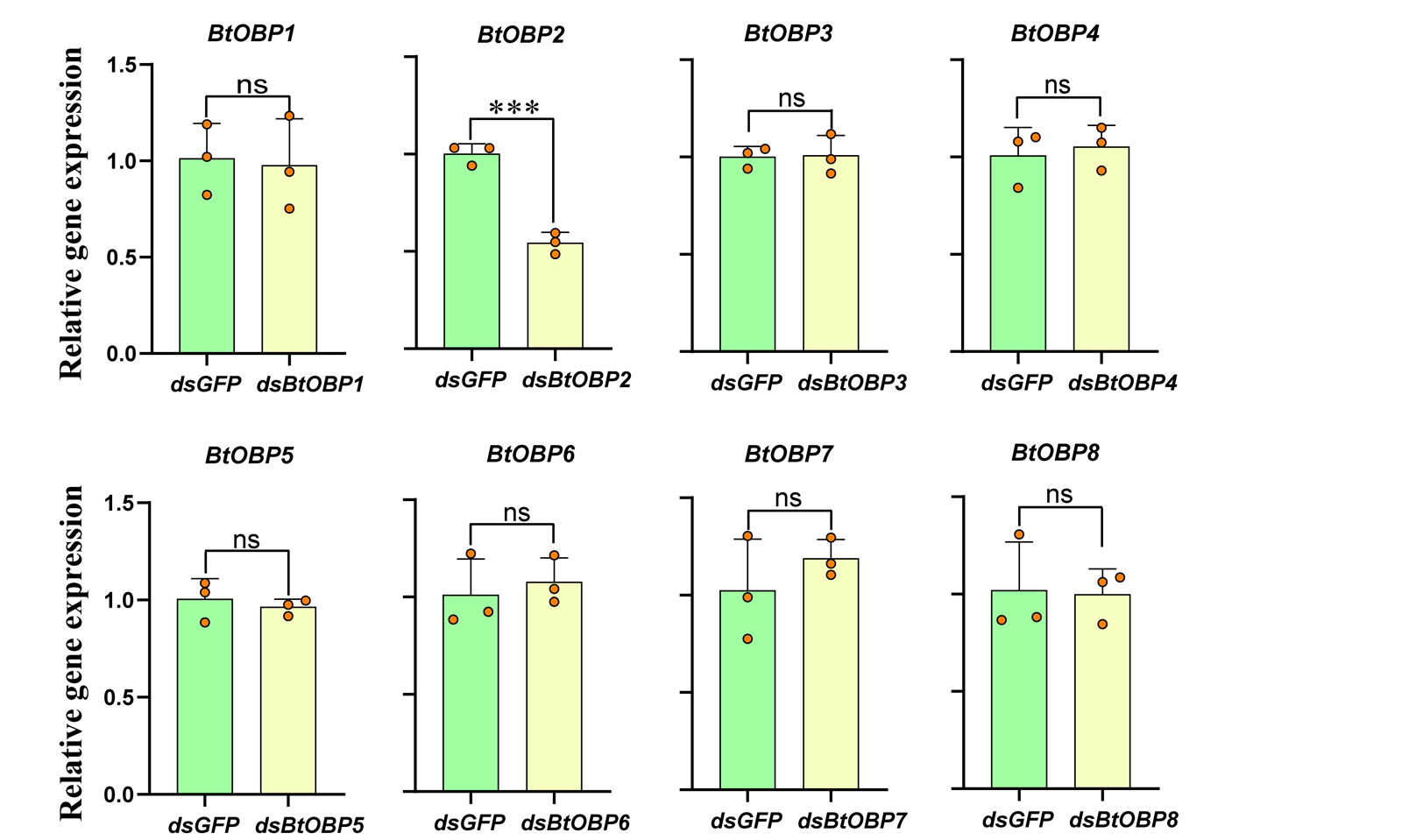


**Figure S8. Relative expression of *BtOBP1-8* in control (*dsGFP*) and *BtOBP2*-silenced (*dsOBP2*) whiteflies.** *****P < 0.001 (n = 3, t-test). Bars in each graph depict mean + SE; orange dots are replicates.**


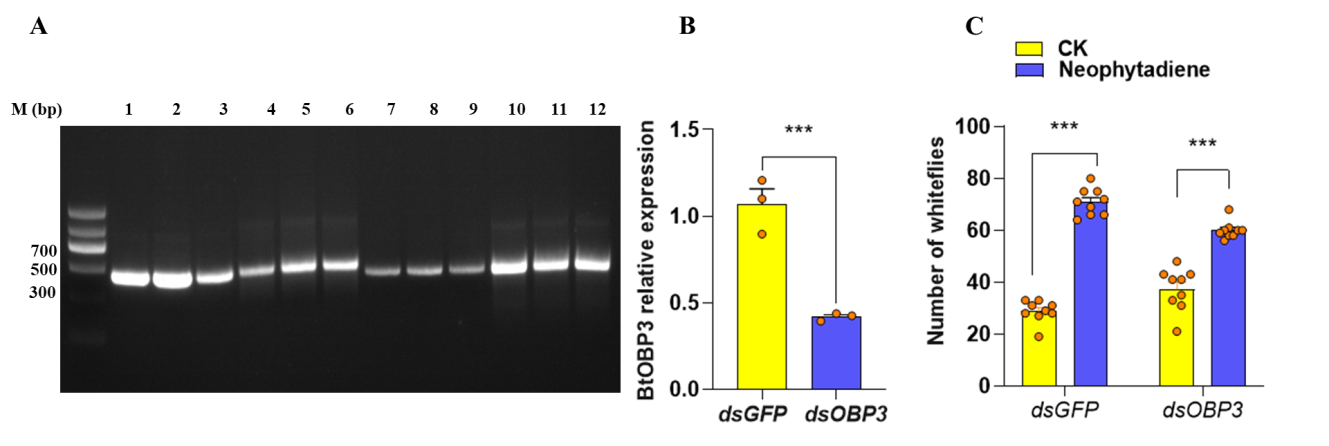


**Figure S9. Whitefly preference to neophytadiene after *BtOBP3* was silenced**.

(A) Synthesis of dsRNAs. M: DNA marker; 1-3: amplification of *dsGFP*; 4-6: *dsGFP*; 7-9: amplification of *dsOBP3;* 10-12:*dsOBP3.* (B) Relative expression of *OBP3* in control (*dsGFP*) and *BtOBP3*-silenced (*dsOBP3*) whiteflies (n = 3, t-test). (C) Whitefly preference for control versus neophytadiene-treated plants; left pair of bars are control whiteflies, right pair of bars are *BtOBP3*-silenced whiteflies (***P < 0.001, n = 9, Two-way ANOVAs).Orange dots are replicates.


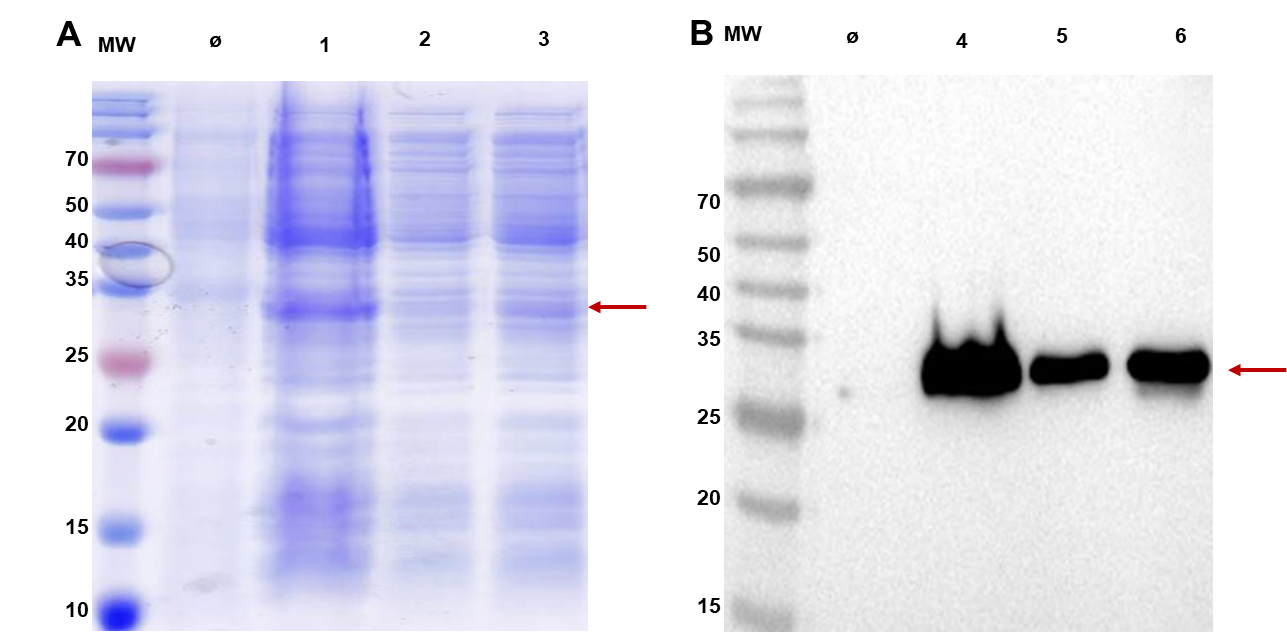


**Figure S10. Expression of the protein BtOBP2.** (A) The expression of the protein BtOBP2 detected by sodium dodecyl sulfate polyacrylamide gel electrophoresis (SDS-PAGE). (B) The expression of the protein BtOBP2 detected by Western blot (WB) analysis using anti His-Tag antibody.

**
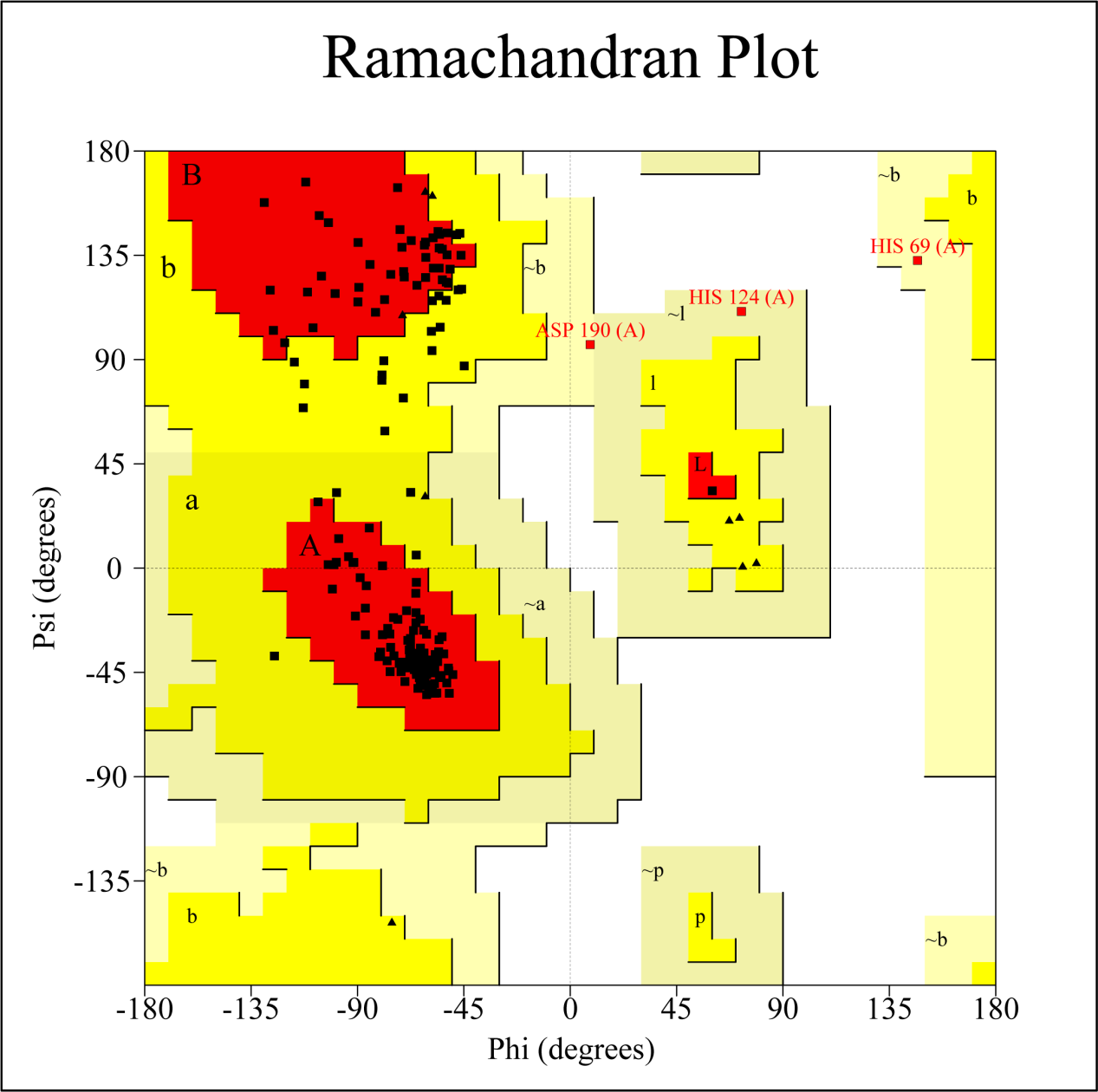
Fig S11. Ramachandran plot of BtOBP2 produced by PROCHECK after homology modeling. [A, B, L] most favored regions; [a, b, l, p] additional allowed regions; [~ a, ~ b, ~ l, ~ p] generously allowed regions; white areas are disallowed regions.** No amino acid residues were in the disallowed region. 9.7% of the amino acid residues were in the allowable region, and 1.9% of the amino acid residues were in the maximum allowable region.


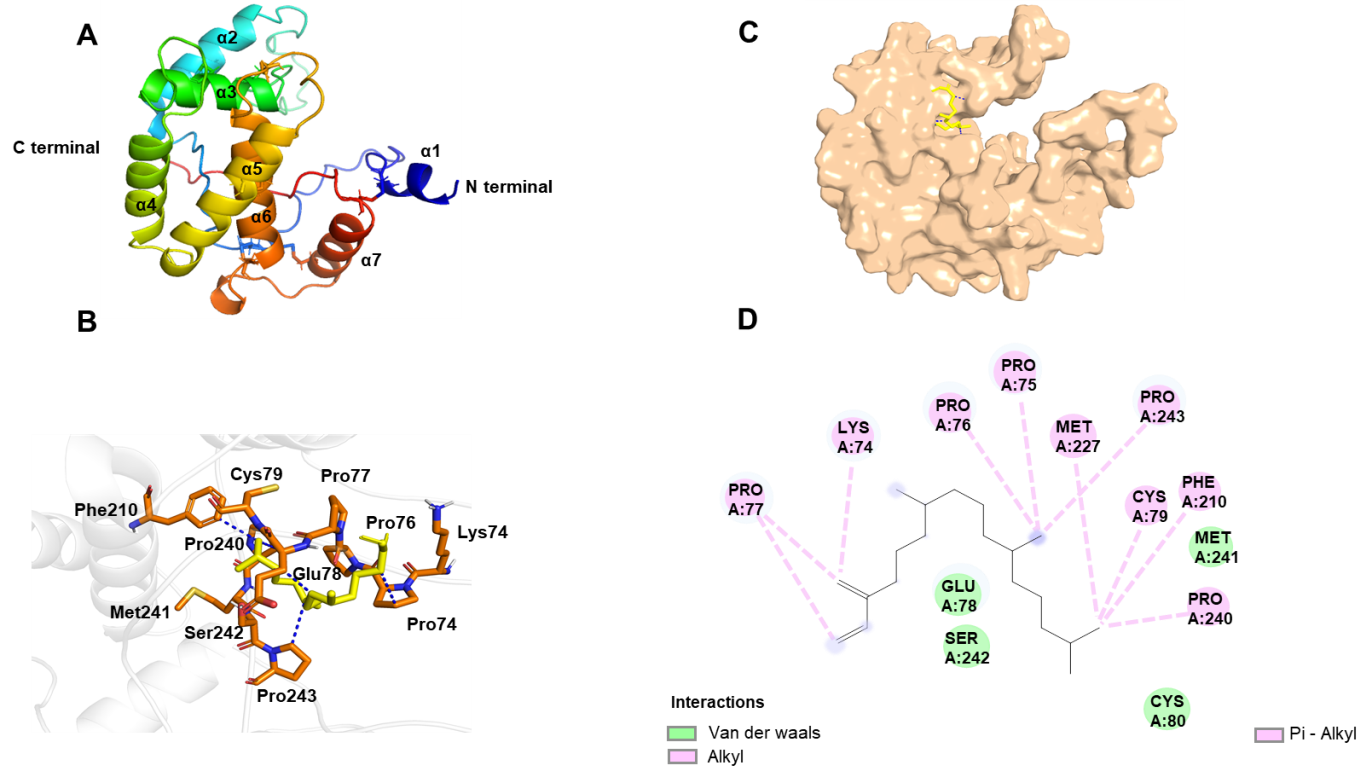


**Figure S12. Homology modeling and molecular docking analysis for BtOBP2.** (A) Predicted the 3D model of *BtOBP2* using AplhaFold. (B) Model diagram of the interaction between neophytadiene and *BtOBP2*. (C) Specific binding sites of *BtOBP2* to neophytadiene; (D) Plan of the interaction between neophytadiene and *BtOBP2*.

Table S1. The main primers and probes used in this study.

| **Primer or Probe name** | **Sequence (5'-3')** | | **Purpose** |
| --- | --- | --- | --- |
| mtCOI1-F | GAATTGACTTATTCCAT | | Whitefly determination |
| mtCOI1-R | CGATCAGACGAATAACCTC | |  |
| P9-F | TCAGCACCAGCTAGCATCGATATGGATCTTGAGGAAATGAT | | cloning and |
| P9-R | GCCTGGAGCGGCCGCGTCGACATGGATCTTGAGGAAATGAT | | plasmid construction |
| P22-F | TCAGCACCAGCTAGCATCGATATGGATCTCACTGGTTGCTT | |  |
| P22-R | GCCTGGAGCGGCCGCGTCGACTTATATA TCACTCCCAAAGA | |  |
| CP-F | TCAGCACCAGCTAGCATCGATATGGAGAACAGTGCTGTTGCA | |  |
| CP-R | GCCTGGAGCGGCCGCGTCGACTTAGCAACCAGTTATCGATGC | |  |
| CPm-F | TCAGCACCAGCTAGCATCGATATGGATGAAAATGAAATCTA | |  |
| CPm-R | GCCTGGAGCGGCCGCGTCGACTCAGAAAAGCTGACCCGTGC | |  |
| P4-F | TCAGCACCAGCTAGCATCGATATGCCTACGGCTGGTATGTT | |  |
| P4-R | GCCTGGAGCGGCCGCGTCGACTCATTTAAATTTCACAAAGA | |  |
| P5-F | TCAGCACCAGCTAGCATCGATATGGGTGATCGTTTTGCTTG | |  |
| P5-R | GCCTGGAGCGGCCGCGTCGACCTAATTAACACGATAAATTT | |  |
| P7-F | TCAGCACCAGCTAGCATCGATATGATTTCCACTTATTTTAC | |  |
| P7-R | GCCTGGAGCGGCCGCGTCGACTTAACGAACTATTGGAGGTT | |  |
| P8-F | TCAGCACCAGCTAGCATCGATATGTTAGTTTGTTTAGTACT | |  |
| P8-R | GCCTGGAGCGGCCGCGTCGACCTACTGAGAAAAGTCTTTTG | |  |
| P27-F | TCAGCACCAGCTAGCATCGATATGGAGGTCGTGTACAATTC | |  |
| P27-R | GCCTGGAGCGGCCGCGTCGACTCAAATATAATAATTTGAGA | |  |
| P59-F | TCAGCACCAGCTAGCATCGATATGGAACCCGTTGATACTTC | |  |
| P59-R | GCCTGGAGCGGCCGCGTCGACTCATTTCCTCAAGATCCATG | |  |
| HSP70-F | TCAGCACCAGCTAGCGGCGCGCCATGAGTATTAAAGCTGGTTT | |  |
| HSP70-R | GCCTGGAGCGGCCGCGTCGACTCAAGAGAAATTTCTGTTTT | |  |
| PI II-F | CCTATTCAAGATGTCCCCGTTC | | q(RT)-PCR |
| PI II-R | GGGCAATCCAGAAGATGG | |  |
| LOX-F | ACTCATCAGCACCGACATCG | |  |
| LOX-R | ACTCTCCAGAAAGAACTCCTGC | |  |
| NPR1-F | ATATAGAATTCCTGCTCCAAAGGATCGGTTA | |  |
| NPR1-R | ATATACTCGAGCAGACAAGTCATCAGCATCCA | |  |
| PR1-F | ATCTCATTGTTACTCACTTGTC | |  |
| PR1-R | AACGAGCCCGACCA | |  |
| ACTIN-F | AGGCAGGATTTGCTGGTGATGATGCT | |  |
| ACTIN-R | ATACGCATCCTTCTGTCCCATTCCGA | |  |
| UBI-F | TCGTAAGGAGTGCCCTAATGCTGA | |  |
| UBI-R | CAATCGCCTCCAGCCTTGTTGTAA | |  |
| CHLH-F | GCTTTGGACCCACAGGCTAT | |  |
| CHLH-R | CTGTGCCAACGACTCTCCAT | |  |
| CHLM-F | AAGAAGGTGCCATTGTATCAG | |  |
| CHLM-R | CCATCCAAACTCTCCAAGTC | |  |
| POR-F | GCATCACATTTGCCTCCCTA | |  |
| POR-R | GAGTTCTTGTTCCAGCTCCAGTAC | |  |
| PAO-F | CGAAATTGGCTTAGACGGCAT | |  |
| PAO-R | ATCTGTCCATCATCTGGCGTT | |  |
| PPH-F | TGAGGTAACAGAACACCCTGC | |  |
| PPH-R | TCATTCGACACCCAGTCAGTG | |  |
| RCCR-F | GTGGAGAATCGTCTTGGAGAGTC | |  |
| RCCR-R | CCGGTGGGTAAGTTGCAGTG | |  |
| OBP1-F | AAGTGCTTGACGGATTATTAC | |  |
| OBP1-R | GCATCATATTATCGCAGTGT | |  |
| OBP2-F | CAACAACCCCTCAACCGACA | |  |
| OBP2-R | TCATGTCAGGTGTCAGAAGGC | |  |
| OBP3-F | CGGTTCAGTTCCAACACTGC | |  |
| OBP3-R | TTGATCTTGTTGCATCGCCG | |  |
| OBP4-F | GTTTCTTGGAGTGCGTTTA | |  |
| OBP4-R | TCATCATCATCAGCCTCTT | |  |
| OBP5-F | AAGTAAAGGCTGTGGATGA | |  |
| OBP5-R | CGAGTAATAGTTGTTGTCTTGA | |  |
| OBP6-F | GTAGCAATACAGGTGGAGA | |  |
| OBP6-R | ATGACACTCTTGACATTAGC | |  |
| OBP7-F | TCGAATCAGATGCAGAGGGTG | |  |
| OBP7-R | TATCCGGGGGACTCATTCCA | |  |
| OBP8-F | TGATGGCGTGTCTTATGA | |  |
| OBP8-R | CTGAGGTTGAGTGCTGTA | |  |
| AD-LHCA4-F | ACCAGATTACGCTCATATGATGGCCACTGTAACAACGCAA | | Y2H |
| AD-LHCA4-R | CTCGAGCTCGATGGATCCCGTTGGAAAATGTTTGGATAATGGT | |  |
| BD-P9-F | CAGAGGAGGACCTGCATATGATGGATCTTGAGG | |  |
| BD-P9-R | CTGCAGGTCGACGGATCCCTTATTTCCTTAAACC | |  |
| Lhca4-mCherry-F | GACGAGCTTTACAAGGGTACC ATGGCCACTGTAACAACGCAA | | Co‐localization |
| Lhca4-mCherry-R | TCTAGTTCATCTAGAGGATCC GTTGGAAAATGTTTGGATAATGGT | |  |
| P9-GFP-F | GACGAGCTGTACAAGGGTACCATGGATCTTGAGG | |  |
| P9-GFP-R | TCTAGTTCATCTAGAGGATCCTTATTTCCTTAAACC | |  |
| P9-Nyfp-F | GGAGTCGACGCACAGGGTACC ATGGATCTTGAGG | | BiFC |
| P9-Nyfp-R | TTCGAGCTCGCCTGGGGATCC TTATTTCCTTAAACC | |  |
| Lhca4-Cyfp-F | F: GGAGTCGACGCACAGGGTACC ATGGCCACTGTAACAACGCAA | |  |
| Lhca4-Cyfp-R | TTCGAGCTCGCCTGGGGATCC GTTGGAAAATGTTTGGATAATGGT | |  |
| Lhca4-FLAG-F | GAACACGGGGGACGAGCTCGGTACCATGGCCACTGTAACAA | | Co-IP |
| Lhca4-FLAG-R | CTTTGTAGTCCATGTCGACTCTAGAGTTGGAAAATGTTTGG | |  |
| P9-1300GFP-F | GAACACGGGGGACGAGCTCGGTACCATGGATCTTGAGGAAA | |  |
| P9-1300GFP-R | CCTTGCTCACCATGTCGACTCTAGATTTCCTTAAACCAAAG | |  |
| Lhca4-Nluc-F | GAACACGGGGGACGAGCTCGGTACCATGGCCACTGTAACAA | | LUC |
| Lhca4-Nluc-R | GGGACGCGTACGAGATCTGGTCGACTTAGTTGGAAAATGTTTG | |  |
| p9-Cluc-F | CTCGTACGCGTCCCGGGGCGGTACCATGGATCTTGAGGAAA | |  |
| p9-Cluc-R | GATGATACGAACGAAAGCTCTGCAGTTATTTCCTTAAACCAA | |  |
| OELhca4-F | CAGTGGTCTCACAACATGGCCACTGTAACAACGCA | | Overexpression |
| OELhca4-R | CAGTGGTCTCATACAGTTGGAAAATGTTTGGATAA | |  |
| F-086-F | TAATACGACTCACTATAGGGTTCAGTGGAGAGGGTGAAGGT | | dsRNA synthesis |
| R-612-R | TAATACGACTCACTATAGGGTGTGTGGACAGGTAATGGTTG | |  |
| dsOBP2-F | TAATACGACTCACTATAGGGGAGGACAAGAAAGCATGGGA | | dsRNA synthesis |
| dsOBP2-R | TAATACGACTCACTATAGGGTTCACCGGCTCCAGATTTAC | |  |
| DsOBP3-F | ATTCTCTAGAAGCTTAATACGACTCACTATAGGGCAATCGAAGGGATGCTGATT | |  |
| DsOBP3-R | ATTCTCTAGAAGCTTAATACGACTCACTATAGGGTCACCAACTTTTTGTTGCCA | |  |
| dsEGFP-F | TAATACGACTCACTATAGGGCACAAGTTCAGCGTGTCCG | |  |
| dsEGFP-R | TAATACGACTCACTATAGGGGTTCACCTTGATGCCGTTC | |  |
| Target 1 | GTTTACCGGATGATCCGTTGAGG | | Knockout |
| Target 2 | TCCATTAAGGTAAGTAGGAGAGG | |  |
| Target 3 | CTGAGCTAGTGAACGGTCGATGG | |  |
| KOLhca4-F | CAGTGGTCTCATGCAGTTTACCGGATGATCCGTTGGTTTTAGAG | |  |
| KOLhca4-R | CAGTGGTCTCAAAACTCGACCGTTCACTAGCTCAGTGC | |  |
| Det-F | GTAAAACGACGGCCAGT | | Knockout |
| Det-R | CCAGAAATTGAACGCCGAAG | | detection |
| Det-KOLhca4-F | CAATCCCAGTCGTCAAAATTCC | |  |
| Det-KOLhca4-R | | GTTCTTAATGTCTTGCCAACGTC |  |

Table S2. Differentially expressed proteins on WT and OELhca4 plants with or without P9.

| Accession | Description | WT+H | OELhca4+H | WT+P9 | OELhca4+P9 |
| --- | --- | --- | --- | --- | --- |
| A0A0U1ZHI0 | Cytochrome b6-f complex subunit 4 OS=Solanum lycopersicum OX=4081 GN=petD PE=3 SV=1 | 0.595936 | 1.167393 | -1.48474 | -0.27859 |
| A0A3Q7F1E4 | PSI-F OS=Solanum lycopersicum OX=4081 PE=3 SV=1 | 0.661161 | 1.182783 | -1.40355 | -0.4404 |
| A0A3Q7GDR0 | Uncharacterized protein OS=Solanum lycopersicum OX=4081 GN=101245163 PE=3 SV=1 | 0.709284 | 1.034733 | -1.54486 | -0.19916 |
| A0A3Q7H7F3 | Uncharacterized protein OS=Solanum lycopersicum OX=4081 PE=3 SV=1 | 0.937933 | 1.024404 | -1.25088 | -0.71145 |
| A0A3Q7HD09 | 23 kDa subunit of oxygen evolving system of photosystem II OS=Solanum lycopersicum OX=4081 GN=544077 PE=3 SV=1 | 0.703919 | 1.005979 | -1.5728 | -0.13709 |
| A0A3Q7HFK3 | Uncharacterized protein OS=Solanum lycopersicum OX=4081 PE=3 SV=1 | 0.82927 | 0.933112 | -1.54776 | -0.21463 |
| A0A3Q7HHU2 | Chlorophyll a-b binding protein, chloroplastic OS=Solanum lycopersicum OX=4081 GN=101268123 PE=3 SV=1 | 0.713451 | 1.067703 | -1.50894 | -0.27221 |
| A0A3Q7I0X4 | Chlorophyll a-b binding protein, chloroplastic OS=Solanum lycopersicum OX=4081 GN=101249002 PE=3 SV=1 | 0.766261 | 1.091778 | -1.42637 | -0.43167 |
| A0A3Q7IAX8 | Chlorophyll a-b binding protein, chloroplastic OS=Solanum lycopersicum OX=4081 GN=101253628 PE=3 SV=1 | 0.485312 | 1.354362 | -1.26478 | -0.57489 |
| A0A3Q7IED7 | PSI-K OS=Solanum lycopersicum OX=4081 PE=3 SV=1 | 0.582901 | 1.218302 | -1.42681 | -0.37439 |
| A0A3Q7IFE9 | Uncharacterized protein OS=Solanum lycopersicum OX=4081 GN=101268297 PE=3 SV=1 | 1.020321 | 0.881391 | -1.3832 | -0.51851 |
| A0A3Q7J1M0 | Plastoquinol--plastocyanin reductase OS=Solanum lycopersicum OX=4081 GN=101243864 PE=3 SV=1 | 0.835673 | 1.078788 | -1.34793 | -0.56653 |
| A0A3Q7J3F7 | Chlorophyll a-b binding protein, chloroplastic OS=Solanum lycopersicum OX=4081 GN=101252151 PE=3 SV=1 | 0.646808 | 1.112263 | -1.51094 | -0.24813 |
| A0A3Q7J9G9 | Ferredoxin OS=Solanum lycopersicum OX=4081 PE=3 SV=1 | 0.676237 | 1.117266 | -1.48245 | -0.31105 |
| A0A7G9M5W1 | Photosystem II CP43 reaction center protein OS=Solanum lycopersicum OX=4081 GN=psbC PE=3 SV=1 | 0.890918 | 1.080054 | -1.20615 | -0.76483 |
| K4CYR3 | Uncharacterized protein OS=Solanum lycopersicum OX=4081 PE=4 SV=1 | 0.367035 | 1.36664 | -1.36421 | -0.36946 |
| P07370 | Chlorophyll a-b binding protein 1B, chloroplastic OS=Solanum lycopersicum OX=4081 GN=CAB1B PE=3 SV=1 | 0.761586 | 1.116658 | -1.39143 | -0.48682 |
| P10707 | Chlorophyll a-b binding protein 1D (Fragment) OS=Solanum lycopersicum OX=4081 GN=CAB1D PE=3 SV=1 | 0.820091 | 1.008118 | -1.47978 | -0.34843 |
| P10708 | Chlorophyll a-b binding protein 7, chloroplastic OS=Solanum lycopersicum OX=4081 GN=CAB7 PE=3 SV=1 | 0.465595 | 1.371685 | -1.24567 | -0.59161 |
| P27524 | Chlorophyll a-b binding protein CP24 10A, chloroplastic OS=Solanum lycopersicum OX=4081 GN=CAP10A PE=3 SV=1 | 0.775563 | 1.136165 | -1.33022 | -0.58151 |
| P27525 | Chlorophyll a-b binding protein CP24 10B, chloroplastic OS=Solanum lycopersicum OX=4081 GN=CAP10B PE=3 SV=1 | 0.948071 | 0.998062 | -1.29812 | -0.64801 |
| Q2MI71 | Cytochrome b6 OS=Solanum lycopersicum OX=4081 GN=petB PE=3 SV=1 | 0.639432 | 1.236542 | -1.32686 | -0.54911 |
| Q2MI75 | Photosystem II CP47 reaction center protein OS=Solanum lycopersicum OX=4081 GN=psbB PE=3 SV=1 | 0.804821 | 1.030619 | -1.46802 | -0.36742 |
| Q2MI83 | Cytochrome b559 subunit alpha OS=Solanum lycopersicum OX=4081 GN=psbE PE=3 SV=1 | 0.521364 | 1.349083 | -1.21707 | -0.65338 |
| Q2MI87 | Cytochrome f OS=Solanum lycopersicum OX=4081 GN=petA PE=3 SV=1 | 0.813087 | 1.037594 | -1.44964 | -0.40104 |
| Q2MIA5 | Photosystem II D2 protein OS=Solanum lycopersicum OX=4081 GN=psbD PE=3 SV=1 | 0.793355 | 1.040028 | -1.46818 | -0.3652 |
| Q2MIC0 | Photosystem II protein D1 OS=Solanum lycopersicum OX=4081 GN=psbA PE=3 SV=1 | 0.741321 | 1.074036 | -1.47738 | -0.33797 |
| Q3SC83 | Cytochrome b559 subunit beta OS=Solanum lycopersicum OX=4081 GN=psbF PE=3 SV=1 | 0.543513 | 1.297995 | -1.32339 | -0.51812 |
| Q672Q6 | Photosystem II oxygen-evolving complex protein 3 OS=Solanum lycopersicum OX=4081 GN=PsbQ PE=2 SV=1 | 0.870538 | 1.062053 | -1.31755 | -0.61505 |
| Q7M1K8 | Chlorophyll a-b binding protein, chloroplastic OS=Solanum lycopersicum OX=4081 GN=101256006 PE=3 SV=1 | 0.877757 | 1.080724 | -1.24734 | -0.71114 |
| A0A3Q7EWQ3 | Ferredoxin--NADP reductase, chloroplastic OS=Solanum lycopersicum OX=4081 GN=101268044 PE=3 SV=1 | 1.270888 | 0.681724 | -1.05922 | -0.89339 |
| A0A3Q7F529 | Uncharacterized protein OS=Solanum lycopersicum OX=4081 GN=101253342 PE=3 SV=1 | 0.657346 | 1.289853 | -0.90886 | -1.03834 |
| A0A3Q7F8X2 | Ferredoxin--NADP reductase, chloroplastic OS=Solanum lycopersicum OX=4081 PE=3 SV=1 | 1.012407 | 0.987515 | -0.99996 | -0.99996 |
| A0A3Q7GAU9 | FAD-binding FR-type domain-containing protein OS=Solanum lycopersicum OX=4081 GN=101262213 PE=4 SV=1 | 0.938674 | 1.054254 | -0.89265 | -1.10028 |
| A0A3Q7GV49 | Protein kinase domain-containing protein OS=Solanum lycopersicum OX=4081 PE=4 SV=1 | 0.930123 | 1.056799 | -1.14198 | -0.84494 |
| A0A3Q7GZ74 | Uncharacterized protein OS=Solanum lycopersicum OX=4081 GN=101263124 PE=3 SV=1 | 0.722944 | 1.215416 | -0.72271 | -1.21565 |
| A0A3Q7HA75 | Chlorophyll a-b binding protein, chloroplastic OS=Solanum lycopersicum OX=4081 PE=3 SV=1 | 0.629863 | 1.311179 | -0.95911 | -0.98193 |
| A0A3Q7HKD5 | Uncharacterized protein OS=Solanum lycopersicum OX=4081 PE=4 SV=1 | 1.207456 | 0.720961 | -1.24957 | -0.67884 |
| A0A3Q7IN40 | 2Fe-2S ferredoxin-type domain-containing protein OS=Solanum lycopersicum OX=4081 GN=101266059 PE=4 SV=1 | 1.119733 | 0.862913 | -0.85691 | -1.12574 |
| A0A3Q7JSZ2 | Chlorophyll a-b binding protein, chloroplastic OS=Solanum lycopersicum OX=4081 GN=101265617 PE=3 SV=1 | 0.976187 | 1.011851 | -1.14743 | -0.84061 |
| A0A3Q7JX44 | Uncharacterized protein OS=Solanum lycopersicum OX=4081 GN=101254882 PE=3 SV=1 | 0.800558 | 1.177505 | -0.89897 | -1.0791 |
| Q2MI72 | Photosystem II reaction center protein H OS=Solanum lycopersicum OX=4081 GN=psbH PE=3 SV=1 | 1.031355 | 0.955955 | -1.14818 | -0.83913 |
| Q2MI93 | ATP synthase subunit beta, chloroplastic OS=Solanum lycopersicum OX=4081 GN=atpB PE=3 SV=1 | 0.904773 | 1.090167 | -0.95853 | -1.03641 |
| Q2MI94 | ATP synthase epsilon chain, chloroplastic OS=Solanum lycopersicum OX=4081 GN=atpE PE=3 SV=1 | 0.972637 | 1.025626 | -1.05177 | -0.9465 |
| Q2MIB2 | ATP synthase subunit a, chloroplastic OS=Solanum lycopersicum OX=4081 GN=atpI PE=3 SV=1 | 0.660619 | 1.283593 | -0.85811 | -1.0861 |
| Q2MIB4 | ATP synthase subunit b, chloroplastic OS=Solanum lycopersicum OX=4081 GN=atpF PE=3 SV=1 | 1.043279 | 0.955729 | -1.00773 | -0.99128 |
| Q2MIB5 | ATP synthase subunit alpha, chloroplastic OS=Solanum lycopersicum OX=4081 GN=atpA PE=3 SV=1 | 0.898474 | 1.096256 | -1.02468 | -0.97005 |
| Q40163 | Photosystem II 10 kDa polypeptide, chloroplastic OS=Solanum lycopersicum OX=4081 GN=PSBR PE=2 SV=1 | 0.961304 | 1.037948 | -0.99438 | -1.00487 |
| A0A3Q7F386 | PSI-F OS=Solanum lycopersicum OX=4081 GN=101245121 PE=3 SV=1 | 0.877428 | 0.745511 | -1.63529 | 0.012354 |
| A0A3Q7FCM5 | Ferredoxin OS=Solanum lycopersicum OX=4081 PE=3 SV=1 | 1.031596 | 0.724771 | -0.21936 | -1.53701 |
| K4BRC3 | ATP-synt_ab domain-containing protein OS=Solanum lycopersicum OX=4081 PE=4 SV=1 | 0.889599 | 0.893884 | -0.25175 | -1.53173 |
| A0A3Q7H2Q4 | Uncharacterized protein OS=Solanum lycopersicum OX=4081 PE=4 SV=1 | 0.244145 | 1.083803 | -1.63455 | 0.306603 |
| P12360 | Chlorophyll a-b binding protein 6A, chloroplastic OS=Solanum lycopersicum OX=4081 GN=CAB6A PE=2 SV=1 | 0.274563 | 1.273799 | -1.51693 | -0.03144 |
| P14279 | Chlorophyll a-b binding protein 5, chloroplastic (Fragment) OS=Solanum lycopersicum OX=4081 GN=CAB5 PE=2 SV=1 | 0.462666 | 1.050217 | -1.63353 | 0.120649 |
| P27489 | Chlorophyll a-b binding protein 13, chloroplastic OS=Solanum lycopersicum OX=4081 GN=CAB13 PE=1 SV=1 | 0.41276 | 1.089647 | -1.62117 | 0.118764 |
